# FRPR-4 Is a G-Protein Coupled Neuropeptide Receptor That Regulates Behavioral Quiescence and Posture in *Caenorhabditis elegans*

**DOI:** 10.1371/journal.pone.0142938

**Published:** 2015-11-16

**Authors:** Matthew D. Nelson, Tom Janssen, Neil York, Kun He Lee, Liliane Schoofs, David M. Raizen

**Affiliations:** 1 Department of Neurology, Perelman School of Medicine, University of Pennsylvania, Philadelphia, Pennsylvania, United States of America; 2 Department of Biology, Saint Joseph’s University, Philadelphia, Pennsylvania, United States of America; 3 Functional Genomics and Proteomics lab, University of Leuven, Leuven, Belgium; Wake Forest University, UNITED STATES

## Abstract

Neuropeptides signal through G-protein coupled receptors (GPCRs) to regulate a broad array of animal behaviors and physiological processes. The *Caenorhabditis elegans* genome encodes approximately 100 predicted neuropeptide receptor GPCRs, but *in vivo* roles for only a few have been identified. We describe here a role for the GPCR FRPR-4 in the regulation of behavioral quiescence and locomotive posture. FRPR-4 is activated in cell culture by several neuropeptides with an amidated isoleucine-arginine-phenylalanine (IRF) motif or an amidated valine-arginine-phenylalanine (VRF) motif at their carboxy termini, including those encoded by the gene *flp-13*. Loss of *frpr-4* function results in a minor feeding quiescence defect after heat-induced cellular stress. Overexpression of *frpr-4* induces quiescence of locomotion and feeding as well as an exaggerated body bend posture. The exaggerated body bend posture requires the gene *flp-13*. While *frpr-4* is expressed broadly, selective overexpression of *frpr-4* in the proprioceptive DVA neurons results in exaggerated body bends that require *flp-13* in the ALA neuron. Our results suggest that FLP-13 and other neuropeptides signal through FRPR-4 and other receptors to regulate locomotion posture and behavioral quiescence.

## Introduction

Neuropeptides modulate multiple homeostatic aspects of animal physiology, including water balance, sexual drive, appetite, and sleep. Neuropeptides affect behavior via their interaction with membrane bound receptors, most of which have seven transmembrane domains and couple to hetero-trimeric G-proteins, which in turn couple to intracellular effector proteins. Recent work in invertebrate model systems has provided insight into the physiological function of neuropeptide signaling pathways *in vivo* [1].

Nematodes, like other animals, contain a large number of neuropeptide and neuropeptide receptors. In the genome of the nematode *Caenorhabditis elegans*, there are over 100 genes predicted to encode neuropeptides, classified as insulin-like, or *ins*, neuropeptide-like, or *nlp*, and FMRFamide-like peptides, or *flp* [2–4], and at least 91 genes predicted to encode neuropeptide receptors [5–7]. While a number of GPCRs have been shown to interact with specific peptides in cell culture systems, in only a few cases have cognate peptide / receptor pairs and the physiological process they control been identified. For example, learning and reproductive behaviors are modulated by NTC-1 acting on NTR-1, a conserved signaling pathway related to mammalian vasopressin/oxytocin [8, 9]. The identification of the NTC-1/NTR-1 signaling pathway in *C*. *elegans* emphasizes the evolutionary conservation of neuropeptide signaling pathways, and suggests that *in vivo* identification of other ligand-receptor pairs in *C*. *elegans* will provide insight into other conserved aspects of animal physiology. This study focuses on a previously unstudied GPCR encoded by the FMRFamide-like peptide receptor-4 gene *frpr-4*, and its potential *in vivo* ligands.

Previously, we have shown that the FLP-13 FMRFamide-like neuropeptides are required for quiescent behavior after environmental exposure to cellular stressors [10], a behavior that enhances recovery from the stress [11]. FMRFa, a *Drosophila* peptide related to peptides encoded by the *C*. *elegans* FLP peptides, signals through its receptor FR to regulate recovery sleep in response to cellular stress [12, 13]. In this study, we provide evidence that FRPR-4, which is a *C*. *elegans* ortholog of *Drosophila* FR, can act both *in vitro* and *in vivo* as a receptor for FLP-13 neuropeptides, and functions specifically in the DVA proprioceptive neuron to regulate body posture.

## Results

### FRPR-4 is a G-protein coupled receptor related to *D*. *melanogaster* FR

Our initial interest in FRPR-4 stemmed from the motivation to identify the mechanism by which FLP-13 neuropeptides promote quiescence of locomotion and feeding in response to cellular stress [10]. In *D*. *melanogaster*, FMRFamide peptides similar to *C*. *elegans flp-13*-derived peptides signal through the FR receptor to regulate an analogous recovery sleep, which occurs in response to heat or infectious stress [12]. Thus, we hypothesized that a homolog of FR in *C*.*elegans* may be a FLP-13 receptor. Phylogenetic analysis of all predicted neuropeptide receptors from *C*. *elegans* and *D*. *melanogaster* showed that FR is related to a group of closely-related paralogous GPCRs, including one encoded by the gene *frpr-4* (S1 Fig). *frpr-4* encodes a receptor of the Rhodopsin class A type, and is predicted to be a neuropeptide receptor [5].

Using 3’-rapid amplification of cDNA ends (3’RACE) [14], we identified three isoforms of *frpr-4*, which differed in their last exon and 3’-untranslated region (UTR) (S2 Fig and S1 Table). We named these isoforms FRPR-4A, B and C. Each isoform contained an apparent 1390-bp retrotransposon flanked by 55-bp terminal inverted repeats in the large 3’ intron. The gene model identified by our cDNA analysis differed at both the 5’ end and 3’ end from the gene model predicted on WormBase (www.wormbase.org). The 5’ end in our experimentally-verified gene model was shorter, and the 3’ end contained two additional exons. The sequence of one of the new 3’ exons identified by our cDNA analysis was highly conserved in the predicted *frpr-4* mRNA from the nematode *Caenorhabditis briggsae* (S3 Fig), supporting the notion that this exon encoded part of the protein.

Based on the similarity between *Drosophila* FR and *C*. *elegans* FRPR-4, we hypothesized that FRPR-4 is activated by FMRFamide-like peptides and may regulate quiescent behavior in *C*. *elegans*.

### FRPR-4 is activated by FLP-13 FMRF-like peptides *in cellulo*

To determine if FLP-13 or other peptides can activate FRPR-4, we used an *in vitro* cell expression system (*in cellulo*). We cloned FRPR-4A (S2 Fig and S1 Table) into a mammalian expression plasmid and transiently expressed the protein in Chinese Hamster Ovary (CHO) cells that also expressed the Ca^2+^ sensitive photoprotein aequorin targeted to the mitochondria and the promiscuous G_alpha16_ subunit. G_alpha16_ causes Ca^2+^ flux in response to receptor activation regardless of the type of G-protein that couples to the receptor *in vivo* [15]. We tested a library of 262 known and predicted *C*. *elegans* neuropeptides at a concentration of 10μM for their ability to elicit a Ca^2+^ flux in cells expressing FRPR-4A. The peptide library contained peptides of the FMRFamide-like peptide (FLP) family as well as other neuropeptide-like proteins (NLPs). Peptides encoded by the genes *flp-5*, *flp-10*, *flp-11*, *flp-13* and *flp-17* were all capable of activating FRPR-4A *in cellulo* at 10μM, suggesting that FRPR-4A is activated by FLPs *in vivo*. Peptides derived from these genes have similar C-terminal endings consisting of an amidated isoleucine-arginine-phenylalanine (IRFa) motif or an amidated valine-arginine-phenylalanine (VRFa) motif, suggesting that the C-terminus of the peptides plays a prominent role in FRPR-4 receptor activation. However, since other peptides containing this motif (e.g. FLP-2, FLP-4) did not activate FRPR-4, other amino acids must confer specificity to the activation. To further explore differences between activating and non-activating peptides, we compared FLP peptides with the (I/V)RF motif that activated FRPR-4A to (I/V)RF peptides that did not activate FRPR-4A (S4 Fig). In general, peptides that activated FRPR-4A were longer (9.4±1.4 amino acids) than peptides that did not activate FRPR-4A (7.9±1.3 amino acids. p = 0.002, Student’s t test). However, we did not identify additional consistent differences between the two peptide groups in either specific amino acids or in types of amino acids (charged, polar, or hydrophobic) outside of the IRF or VRF motif.

**Fig 1:**
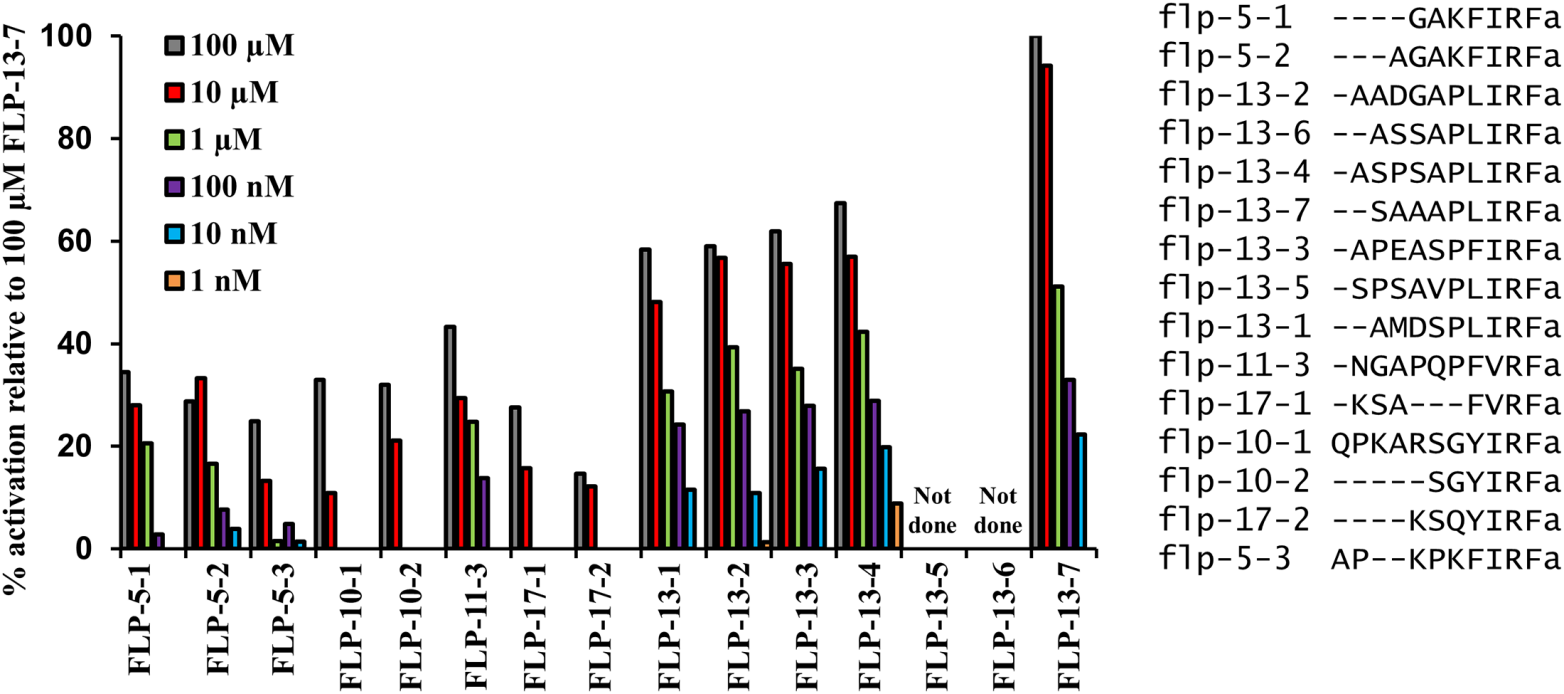
FLP-13 and other FMRFamide-like peptides activate FRPR-4A in a mammalian cell-culture system. Among 262 *C*. *elegans* neuropeptides, peptides encoded by the genes *flp-5*, *flp-10*, *flp-11*, *flp-13* and *flp-17* activate FRPR-4, with *flp-13* derived peptides eliciting the largest amplitude responses. The magnitude of aequorin response to the peptides presented at concentrations ranging from 1 nM to 100 μM and is shown relative to the magnitude of the response to the FLP-13-7 peptide.

**Fig 2:**
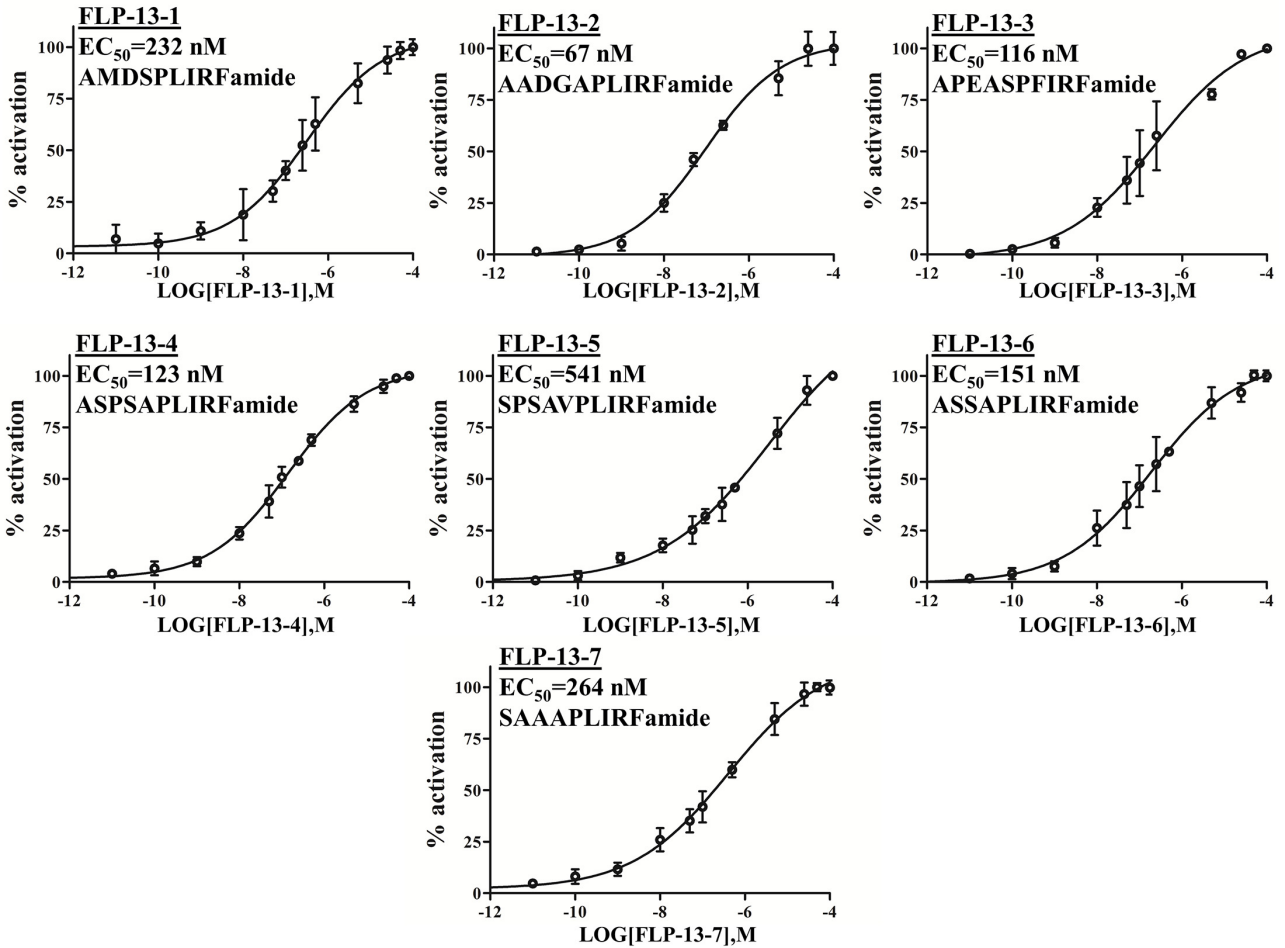
FRPR-4A is activiated by FLP-13 peptides *in cellulo*. Effect of the seven FLP-13 peptides (FLP-13a to g) on the intracellular Ca^2+^ production in CHO/mtAEQ/G_alpha16_ cells expressing the full-length FRPR-4A. Data are presented as means ± s.e.m. percent of maximal activation (N = four trials per experiment).

The response amplitude elicited by these FLP peptides was variable. Peptides encoded by the genes *flp-5*, *flp-11*, and *flp-13* elicited FRPR-4 dependent Ca^2+^ flux at concentrations of 100 nM or lower, whereas peptides encodes by *flp-10* and *flp-17* elicited a detectable Ca^2+^ signal only at a concentration of 10 μM or greater. In general, neuropeptides are believed to signal *in vivo* at concentration in the picomolar to nanomolar range [16, 17]. At the lowest peptides concentrations, *flp-13*-derived peptides elicited the largest magnitude FRPR-4-dependent Ca^2+^ signals (Fig 1), suggesting that they are the most potent activators of FRPR-4A. We then tested a range of concentrations for each of the seven *flp-13* derived peptides and found that they activated FRPR-4 with EC_50_ values ranging from 67 nM to 541 nM (Fig 2). These values are similar to the those measured for activation of peptide dispersing factor (PDF) receptor by its ligand PDF, a well characterized ligand receptor pair; the PDF/PDFR EC_50_ values range from 34 nM to 361 nM [18]. Our *in cellulo* results suggest that peptides encoded by the genes *flp-5*, *flp-11*, and *flp-13* are the best candidates for being *in vivo* FRPR-4A ligands. Because *flp-13* was the strongest *in cellulo* activator of FRPR-4A and because of our initial interest in FLP-13, we focused our efforts on *in vivo* interactions between *frpr-4* and *flp-13*.

### FRPR-4 regulates behavioral quiescence

We reasoned that if FRPR-4 were an *in vivo* receptor for somnogenic FLP-13 neuropeptides, then manipulating *frpr-4* activity should have similar effects to those observed when we manipulated *flp-13* gene activity. That is, overexpression of *frpr-4* might promote quiescence and loss-of-function of *frpr-4* might produce defects in quiescence [10]. The phenomenon of the same phenotype arising from increased activity of the neuropeptide or its receptor has been previously observed during genetic dissection of the *C*. *elegans* egg-laying circuit [19] and social feeding behaviors [20]. To test whether *frpr-4* can induce quiescence in normally active animals, we made transgenic animals expressing extra copies of the genomic region that included the *frpr-4* coding region as well as 5 kb of DNA upstream of the start site of translation and the 3’UTR we had identified using 3’RACE (see above). Our aim was to increase the copy number of FRPR-4 in cells where it is normally expressed, and, by doing so, increase FRPR-4 signaling and thus amplify its physiological roles.

**Fig 3:**
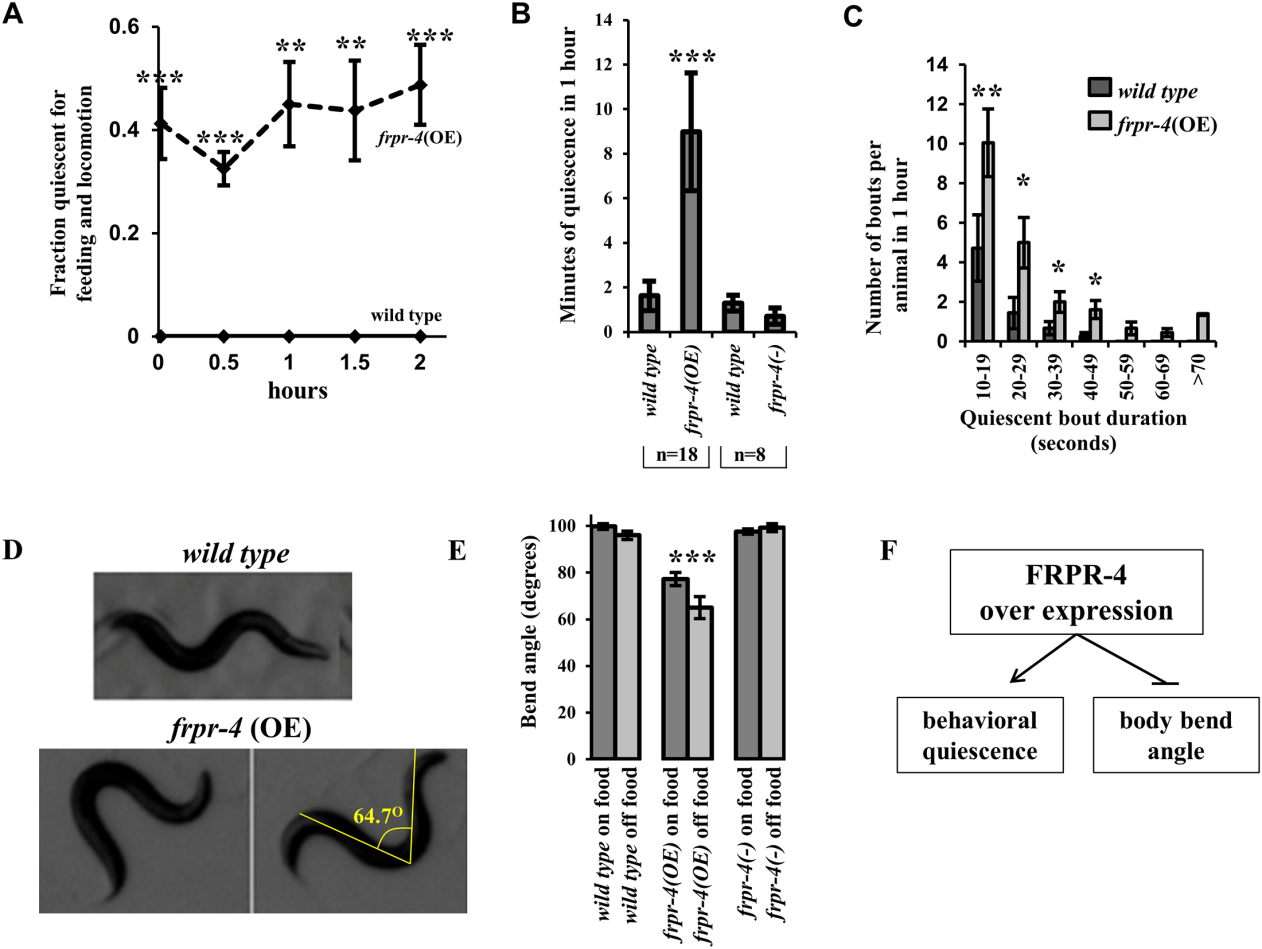
Overexpression of *frpr-4* induces behavioral quiescence and a decreased body bend angle during locomotion. (**A**) A fraction of animals expressing an integrated multi-copy array of the *frpr-4* gene display behavioral quiescence (no feeding or locomotion) when inspected at half-hour intervals over a two-hour period (N≥69, Fisher’s exact test, ***p < .0005). (**B**) Locomotion quiescence of one day old adult animals measured using machine vision analysis. In each experiment, pairs of worms were observed, one of each of the two genotypes grouped in a bracket. Animals expressing the integrated multi-copy array of *frpr-4*, *qnIs195*, are more quiescent than wild-type animals (N = 18, Student’s t-test, ***p < .001). There is no significant difference (p>0.05) between wild-type and *frpr-4*(*ok2376*) worms. (**C**) *frpr-4* over-expressing animals display more frequent bouts and longer-duration bouts. (N = 18, Wilcoxon rank sum test, **p < .005, *p < .05). (**D**) *frpr-4* over-expressing animals display exaggerated body bends in comparison to wild-type animals. A line is drawn from the peak of one bend to the peak of the bend on the opposite side of the animal and the angle formed by the two lines was measured using imageJ software. Animals that over-express *frpr-4* have a significantly reduced bend angle, both on and off food (N>10, Student’s t-test, ***p < .001). (**E**) A summary of *frpr-4* over-expressing phenotypes.

We observed a cohort of adult animals housed on a lawn of bacterial food on an agar surface every 30 minutes. We considered an animal quiescent if we could discern no movements of its body or of its feeding organ, the pharynx, for 15 seconds. Wild-type animals were nearly continually active under these cultivation conditions and thus had only brief pauses of movement and feeding (S1 Movie, **left well**). In contrast, animals carrying multiple transgenic copies of the *frpr-4* gene showed spontaneous bouts of behavioral quiescence (Fig 3A and S1 Movie, **right well**). At every observation time point, up to 40% of animals carrying an integrated multi-copy transgene of FRPR-4 but 0% of wild-type animals was quiescent (Fig 3A). Four strains carrying extrachromosomal transgenes with multiple *frpr-4* copies showed similar effects (S5 Fig). Therefore, *frpr-4* overexpression promotes spontaneous bouts of behavioral quiescence.

To corroborate our assessments of quiescence, we used a machine vision approach to measure total quiescence as well as bout frequency and duration [21, 22]. We measured quiescence in pairs of animals, consisting of one animal overexpressing *frpr-*4 and one control wild-type animal. *frpr-4* over-expressing animals had increased total quiescence (Fig 3B). The increased quiescence can be explained by an increased number of quiescent bouts (21 ± 4 in *frpr-4* over-expressing adults; 7 ± 3 in wild-type adults; p<0.001, Wilcoxon Rank Sum Test), as well as an increased average bout duration (20 ± 3 seconds in *frpr-4* over-expressing animals; 13 ± 1 in wild-type animals; p<0.05) (Fig 3C). This analysis suggests that *frpr-4* promotes both the induction and maintenance of the quiescent behavioral state. Quiescent animals moved when mechanically stimulated (S2 Movie), indicating that they were not paralyzed or injured.

As a complementary approach to studying the effects of over-expressing *frpr-4*, we studied the effects of a loss of *frpr-4* function on stress-induced quiescence. The *ok2376* allele contains a 1540 nucleotide deletion, which removes approximately 300 nucleotides of the *frpr-4* promoter as well as the first two exons of the *frpr-4* gene (S2 Fig). This deletion is predicted to make a truncated protein lacking three N-terminal transmembrane domains. However, because there are potentially alternative start sites for translation, *ok2376* may retain four C-terminal transmembrane domains as well as the intracellular C-terminal domain.

**Fig 4:**
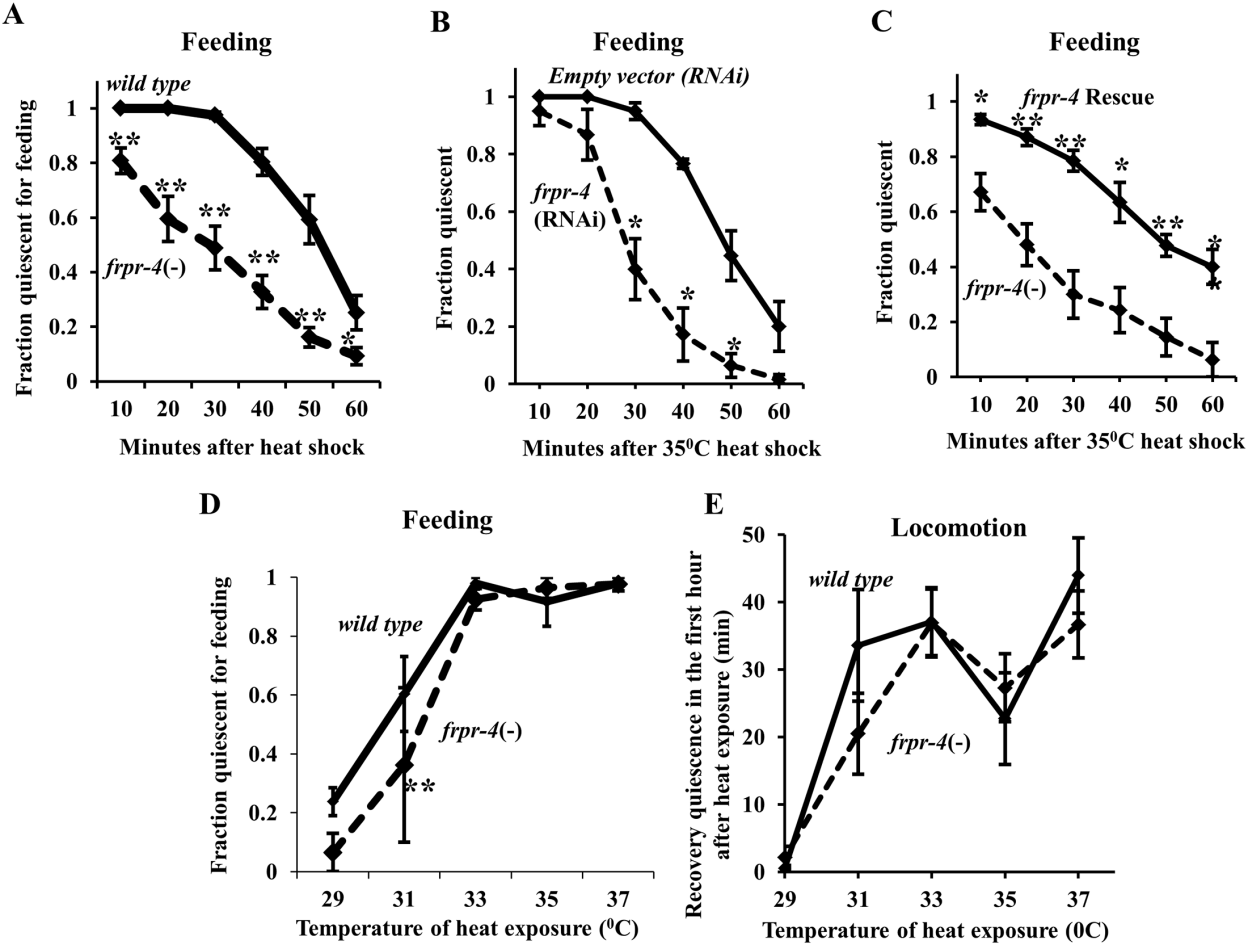
FRPR-4 is partially required for the feeding quiescence response to heat shock. Reducing *frpr-4* function by mutation (**A**) or by RNA interference (**B**) impairs the feeding quiescence response to a 30-minute 35°C heat shock (Protocol 1 (See methods); Student’s t-test, average of 10 trials, N≥20 worms per trial, *p < .05, **p < .005). (**C**) A fosmid containing the wild-type *frpr-4* gene restores in *frpr-4(ok2376)* mutants the feeding quiescence response to a 30-minute 35°C heat shock (Protocol 1 (See methods)) but not at other temperatures (Student’s t-test, average of 4 trials for each temperature, N≥20 worms per trial, **p < .005.) (**D**) *frpr-4*(*ok2376*) animals suppress the feeding quiescence in response to a 30-minute 33°C heat shock but not at the other temperatures tested (Student’s t-test, average of 3 trials, N≥20 worms per trial, **P < .005). (**E**) *frpr-4*(*ok2376*) worms display normal locomotion quiescence in response to heat stress at all temperatures tested (Average of 2 trials, 12 worms per trial).

FLP-13 neuropeptides released from the ALA neuron are partially required for the sleep-like quiescent behavior that occurs following cellular stress induction [10]. Thus, if *frpr-4* were a FLP-13 receptor, then *frpr-4* mutants should also be deficient in this stress-induced quiescent response. We observed a small but significant reduction in the fraction of *frpr-4*(*ok2376*) animals that were quiescent during recovery from a 30 minute 35°C heat exposure (see methods –protocol 1) (Fig 4A). Reduction of *frpr-4* function via RNA interference (RNAi) had a similar attenuating effect on heat-induced feeding quiescence under the same conditions (Fig 4B), suggesting that *frpr-4*(*ok2376*) is a reduction-of-function allele. Transgenic expression of a fosmid containing the *frpr-4* gene restored the heat-induced feeding quiescence of *frpr-4(ok2376)* mutant animals to that of wild-type animals (Fig 4C).

Previously, we found that worms are highly sensitive to differences in exposure temperature: small differences in heat exposure temperature resulted in large differences in behavioral quiescence following the exposures [10]. We therefore assessed the animals’ quiescent response to a range of temperature exposures. To insure that the duration of temperature exposure was the same in all animals, we placed the animals on pre-heated plates to start the 30-minute temperature exposure, and removed them from the heated plates to complete the temperature exposure (see methods –protocol 2) [10]. Similar to our prior observations, the magnitude of the recovery quiescent response increased with higher exposure temperatures. Following exposure to 31 degrees Celsius but not following exposures to other temperatures, *frpr-4* mutants showed a small defect in feeding quiescence (Fig 4D). In contrast to this defect in feeding quiescence, no exposure temperature resulted in a locomotion quiescence defect (Fig 4E). While the effects of removing *frpr-4* function on stress-induced feeding quiescence was statistically-significant, it was smaller than the effects of removing *flp-13* function [10], suggesting that other receptors are contributing to the quiescence. Unlike the case of *flp-13*, whose mRNA is induced by heat shock [10], *frpr-4* mRNA was not changed by heat shock (S6 Fig).

Because FRPR-4 was activated by FLP-13 peptides *in cellulo*, *frpr-4* overexpression promoted quiescent behaviour, and *frpr-4* mutants had a defect in stress-induced quiescence, we hypothesized that FLP-13 peptides are the activators of the FRPR-4 receptor *in vivo*. Based on this hypothesis, we predicted that the elevated quiescence phenotype of animals over expressing *frpr-4* would be attenuated by removing the *flp-13* gene. We additionally predicted that *frpr-4* loss-of-function would attenuate the quiescent phenotype observed with overexpression of *flp-13* [10]. To test these predictions, we over-expressed *frpr-4* in the *flp-13(tm2427)* null mutant background and over-expressed *flp-13* in the *frpr-4(ok2376)* mutant background. Contrary to our predictions, the elevated locomotion quiescence (S7A Fig) and the quiescence bout frequency (S7B Fig) of *frpr-4* over-expressing animals were not significantly reduced by the *flp-13(tm2427)* mutation. In addition, the *frpr-4(ok2376)* mutation did not attenuate the quiescence induced by FLP-13 overexpression (S7C Fig). Together, these experiments suggest that (1) FRPR-4 is not the (sole) *in vivo* receptor for FLP-13 peptides, and (2) FLP-13 peptides are not the (sole) ligands for all three FRPR-4 receptor isoforms or FRPR-4 may have ligand-independent activity.

### FRPR-4 affects body posture by acting in the DVA neuron in a *flp-13* dependent fashion

In the course of the experiments in which we closely observed animals for quiescent behaviour, we noted a body posture phenotype of *frpr-4* over-expressing animals. *C*. *elegans* worms crawl on an agar surface with a wave of posteriorly directed ventral/dorsal body bends made in the vector perpendicular to the agar surface. We noted that body bends made by *frpr-4* over-expressing animals were deeper than those made by wild-type animals. The deeper bends were the result of a reduction in the angle produced by the body bends during locomotion (Fig 3D). To quantify this phenotype, we measured the angle produced by the body bend in first day adult worms (Fig 3D). To reduce the chance that the altered body posture might be explained by difference in posture between quiescent and active worms, we measured the body angle bends only during active bouts. *frpr-4* over-expressing animals had a significantly reduced bend angle compared to wild-type animals during movement both in the presence and in the absence of food (Fig 3E, S3 and S4 Movies). Thus, FRPR-4 is capable of promoting reduced body bend angles and thus exaggerated body bends (Fig 3F).

**Fig 5:**
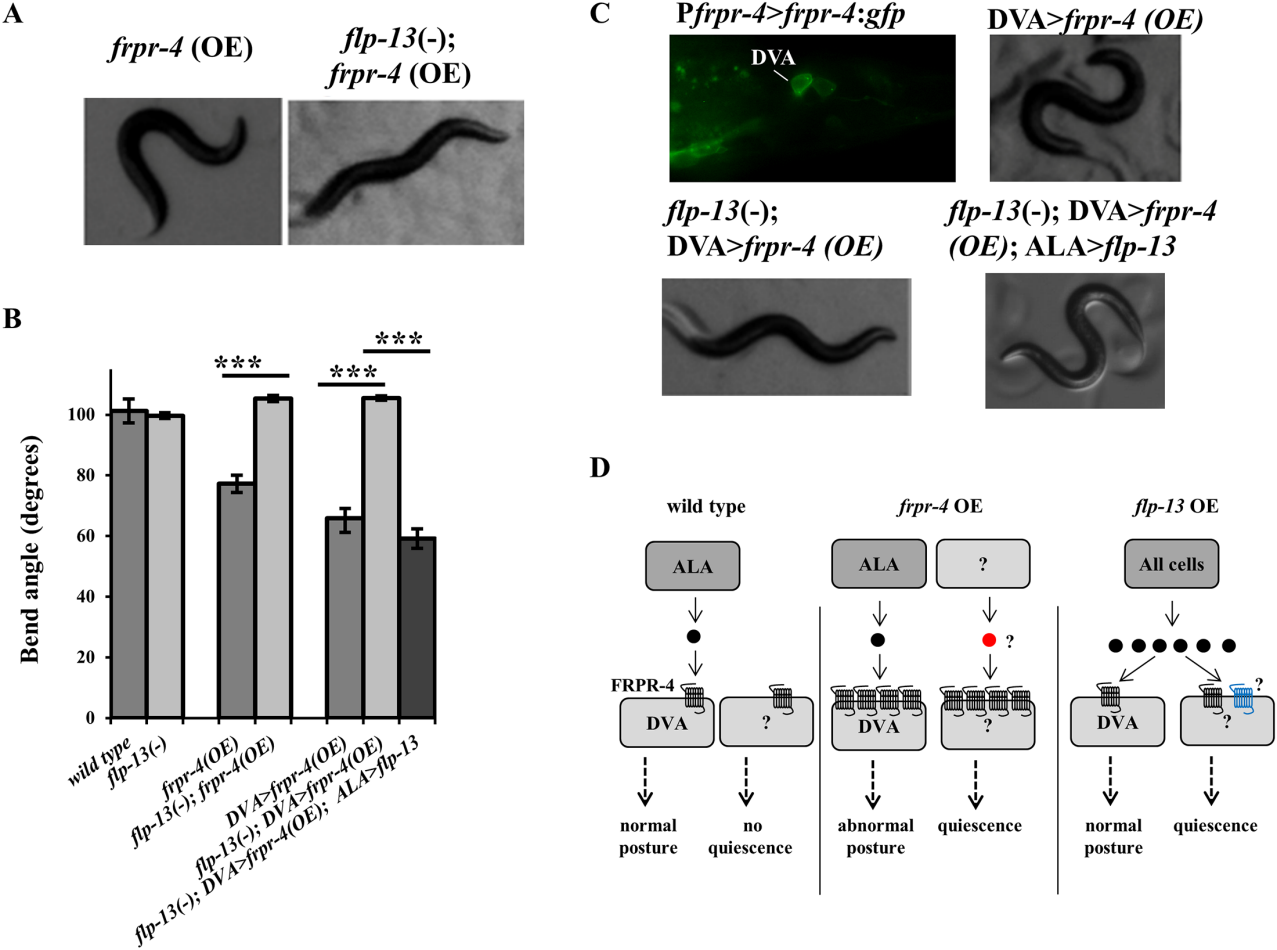
FLP-13 signals through FRPR-4 in DVA to regulate posture. (**A-B**) Overexpression of *frpr-4* from its endogenous promoter reduces the animals bend angle. The *flp-13*(*tm2427*) deletion suppresses this phenotype and expression of *flp-13* in the ALA neuron restores this phenotype (Student’s t-test, N>10, ***P < .001). (**C**) A strain carrying an *frpr-4*:*gfp* translational reporter shows GFP localization to the membrane of the DVA neuron. Overexpression of *frpr-4* in the DVA neuron using the promoter from the gene *twk-16* results in a decrease in bend angle, which is suppressed by the *flp-13*(*tm2427*) deletion and then restored by reinstating *flp-13* in the ALA neuron. (**D**) A model of the effects of *frpr-4* or *flp-13* overexpression on posture and behavioral quiescence. Black circle denote FLP-13 peptides; red circle denotes an unknown peptide. Black seven-transmembrane-domain receptors denote FRPR-4. Blue seven-transmembrane-domain receptors denote an unknown receptor mediating the quiescent effects of FLP-13 peptides.

Although *flp-13* was not required for the quiescence-inducing effects of *frpr-4* overexpression, we observed that the exaggerated body bend posture induced by *frpr-4* overexpression was absent in the *flp-13*(*tm2427*) mutants (Fig 5A and S5 Movie). To quantify this suppression, we again measured the angle produced by the body bends. The *flp-13*(*tm2427*) mutation suppressed the *frpr-4*-overexpression bend phenotype (Fig 5B).

A similar body bend phenotype has been reported in animals that had abnormal activity of the DVA neuron [23]. We therefore hypothesized that FRPR-4 functions in the DVA neuron to alter its activity. To determine if *frpr-4* is expressed in the DVA neuron, we constructed a fluorescent translational reporter that contained >5kb of *frpr-4* upstream regulatory DNA and the entire coding sequence of FRPR-4A, replacing the stop codon with a sequence encoding green fluorescent protein (S2 Fig). We observed broad expression of the *frpr-4*::*gfp* translational reporter during both larval development and adulthood; expression was in body wall muscles, pharyngeal muscles, and neurons (S8 Fig). We observed membrane localization of the green fluorescence, as would be expected for a GPCR. We observed spontaneous bouts of behavioral quiescence during the adult stage in two transgenic lines (S5 Fig), suggesting that the translational reporter protein was functional. To facilitate identification of cells expressing *frpr-4*, we also generated transcriptional reporters in which *gfp* containing a nuclear localization signal was expressed under the control of the 5kb of DNA immediately upstream of the *frpr-4* start site of translation (S8 Fig). As in the case of the translational reporter, we observed broad expression of the transcriptional reporter. Using the transcriptional reporter, we identified *frpr-4* expression in the paired RIA neurons (S8 Fig), which have been implicated in the regulation of locomotion quiescence [24]; the paired I1 pharyngeal neurons, which regulate pharyngeal pumping rate [25] and connect the pharyngeal and somatic nervous systems [26]; the AVE neurons (S8 Fig), command interneurons that regulate locomotion [27] and are post-synaptic to the quiescence-generating ALA neuron [28], and the PVM neurons (S8 Fig), which are mechanosensory [29]. Additionally, as with our translational reporters, we saw bright expression in the DVA neuron (Fig 5C).

To determine if the *frpr-4*-induced body bend phenotype was explained by its activity in the DVA neuron, we used the DVA-specific *twk-16* promoter [30, 31] to over-express *frpr-4*. These animals, like those over-expressing *frpr-4* under the control of its endogenous promoter, showed the exaggerated body bend phenotype (Fig 5B and 5C and S6 Movie), consistent with the notion that *frpr-4* affects posture via its activity in the DVA neuron. The *flp-13*(*tm2427)* mutation suppressed the body bend phenotype of animals over-expressing *frpr-4* in the DVA neuron (Fig 5 and S7 Movie). These genetic interactions between *frpr-4* and *flp-13* together with our *in cellulo* analyses suggest that FRPR-4 is an *in vivo* FLP-13 receptor.

We previously showed that the ALA neuron releases FLP-13 peptides to induce quiescence in response to cellular stress [10]. We hypothesized that FLP-13 peptides are released from ALA to regulate body bend amplitude. To test this hypothesis, we restored *flp-13* in the ALA neuron in the *flp-13*(*tm2427*); DVA>*frpr-4*(OE) background. Expression in ALA was accomplished by expressing the genomic sequence of *flp-13* under the control of the *ida-1* promoter [32]. Restoration of *flp-13* in ALA did indeed result in a re-emergence of the exaggerated bend posture phenotype (Fig 5B and 5C and S8 Movie). These data suggest that FLP-13 is released from ALA to signal through FRPR-4 in the DVA neuron to regulate posture during locomotion.

## Discussion

Using a combination of cell culture experiments and genetic analyses, we provide evidence that FRPR-4 is a G-protein coupled receptor that is activated by IRFamide and VRFamide neuropeptides, and that it can promote both behavioral quiescence and exaggerated body bends. In addition, we show an *in vivo* genetic interaction between *frpr-4* and the P(F/L)IRFamide encoding gene *flp-13*.

While overexpression of either *flp-13* or *frpr-4* induces quiescence, the quiescent phenotype caused by either gene is not dependent on the presence of the other gene (S7 Fig). In addition, *frpr-4* mutants have only a small defect in stress-induced feeding quiescence (Fig 4). Are these results consistent with FRPR-4 being a receptor for FLP-13 *in vivo* to promote quiescent behavior? One possibility is that FLP-13 neuropeptides act through other receptors in addition to FRPR-4, and conversely, that FRPR-4 is activated by other neuropeptides in addition to the FLP-13 neuropeptides. Our *in cellulo* data are consistent with this notion, since several neuropeptides activate FRPR-4. In addition, FRPR-4 has five closely-related paralogs (S1 Fig), which could conceivably act as alternative receptors to FLP-13 peptides. There is precedence for genetic redundancy within neuropeptide signalling pathways. Suppression of the egg-laying defective phenotype caused by increased activity of the EGL-6 GPCR is accomplished by removal of both genes encoding neuropeptide ligands for this receptor; removal of one gene does not fully suppress the phenotype [19]. In addition, we identified three isoforms of FRPR-4 (S2 Fig and S1 Table), which are primarily different at their C-termini. Our *in cellulo* analyses tested only the FRPR-4A isoform, but it is possible that the B and C isoforms have different affinities for FLP-13, or may be activated by different ligands.

With respect to the body posture phenotype, we can draw firmer conclusions regarding FLP-13/FRPR-4 interactions. FRPR-4 overexpression specifically in the DVA neuron causes exaggerated body bends, which require *flp-13* expressed in the ALA neuron. This genetic interaction, coupled with the *in cellulo* interactions between FLP-13 peptides and FRPR-4, supports the model that FLP-13 released from ALA acts directly on FRPR-4 in the DVA neuron to modulate posture.

How do we reconcile this model with our observations that neither a *flp-13* loss-of-function mutation nor a *frpr-4* loss-of-function mutation affects body posture and that *flp-13* overexpression does not mimic the deeper body bend posture of *frpr*-4 over expressing animals? We propose that normally, the amount of expressed FRPR-4 receptor sets a limit on signaling via this pathway. With *frpr-4* expression under typical cultivation conditions, signaling is too low to affect body posture (Fig 5D **–left panel**). Reducing FRPR-4 signaling yet further, by removing the gene or by removing its ligand FLP-13, would have no effect on body posture, consistent with our observations. However, increased *frpr-4* receptor expression in DVA causes increased signaling in DVA to affect body posture. And, while *flp-13* is required for the posture effects of *frpr-4* overexpression, it is not required for the quiescence phenotype, which must be regulated by additional unidentified peptides or by ligand-independent activity (Fig 5D **–middle panel**). Finally, we propose that because of the limiting effects of wild type *frpr-4* expression, *flp-13* overexpression does not induce a body posture phenotype, but does induce quiescence in an *frpr-4*-independent fashion, suggesting that one or more unidentified FLP-13 receptors exist (Fig 5D **–right panel**).

The strong phenotype observed with *frpr-4* multi-copy overexpression suggests a strategy for identifying *in vivo* roles of other neurotransmitter receptor GPCRs. If a receptor shows low expression and signaling under typical laboratory cultivation conditions, the phenotypic difference between absence and presence of the gene may be difficult to detect. Consistent with this notion, in a systematic study examining the phenotypic consequences of reduction of function of neurotransmitter receptor GPCRs, only approximately 15 (of over 60 tested) resulted in a discernible phenotype [7]. In contrast, the difference between multiple copy expression of the gene and the wild-type, low expressing, condition, may be far more apparent, and lead to specific hypotheses regarding the normal function of the gene, which can then be tested using fine phenotypic analysis of the loss-of-function mutants.

## Materials and Methods

### Animal husbandry and strains

Animals were cultivated on NGM agar and fed the OP50 *E*.*coli* derivative strain DA837 [33]. The following strains were used in this study: **N2** (Bristol), **EG4322** *ttTi5605 II; unc-119(ed3)III*, **TM2427** *flp-13*(*tm2427*)IV, **RB1837** *frpr-4*(*ok2376*)II, **NQ291** *unc-119*(*ed3*)III; *qnEx155*[*frpr-4*(+), P*myo-2>mCherry*; *unc-119*(+)], **NQ308** *unc-119*(*ed3*)III; *qnEx195*[*frpr-4*(+), P*myo-2>mCherry*, *unc-119*(+)], **NQ385** *frpr-4*(*ok2376*)II, **NQ408** *unc-119*(*ed3*)III; *qnEx196*[*Pfrpr-4>frpr-4*:*gfp*; P*myo-2>mCherry*; *unc-119*(+)], **NQ459** *unc-119*(*ed3*)III; *qnEx233*[*Pfrpr-4>frpr-4*:*gfp*; *Pglr-3>mCherry*, *unc-119*(+)], **NQ460** *unc-119*(*ed3*)III; *qnEx234*[*Pfrpr-4>frpr-4*:*gfp*: *frpr-4* 3’UTR*; Pglr-3>mCherry*; *unc-119*(+)], **NQ465** *unc-119*(*ed3*)III; *qnEx248*[P*frpr-4*>NLS:*gfp*; *Pglr-3>mCherry*; *unc-119*(+)], **NQ480** *qnIs195*[*frpr-4*(+); P*myo-2>mCherry*; *unc-119*(+)], **NQ588** *flp-13*(*tm2427*)IV; *qnIs195*[*frpr-4*(+), P*myo-2>mCherry*,*unc-119*(+)], **NQ601** *flp-13*(*tm2427*)IV;*qnEx310*[P*ida-1*>*flp-13*,P*rab-3>mCherry*], **NQ602** *flp-13*(*tm2427*)IV (Outcrossed to N2 3 times), **NQ648** *qnEx347*[P*frpr-4*:NLS:*gfp*,P*opt-3*:mCherry], **NQ743** *frpr-4*(*ok2376*) *IV*; *qnEx405*[*frpr-*4(+)(FOSMID-WRM0630bE03,P*myo-2*>*gfp*], **NQ744** *frpr-4*(*ok2376*)*IV*; *qnEx406*[*frpr-*4(+)(FOSMID-WRM0630bE03,P*myo-2*>*gfp*], **NQ745** *frpr-4*(*ok2376*) *IV*; *qnEx407*[*frpr-*4(+)(FOSMID-WRM0630bE03,P*myo-2*>*gfp*], **NQ756** *qnEx415*[P*twk-16>frpr-4*,P*twk-16>frpr-4*:*gfp*, *myo-2>mCherry*], **SJU20** *flp-13*(*tm2427*)IV; *qnEx415*[P*twk-16>frpr-4*,P*twk-16>frpr-4*:*gfp*, *myo-2>mCherry*], **SJU47** *flp-13*(*tm2427*)IV; *qnEx415*[P*twk-16>frpr-4*,P*twk-16>frpr-4*:*gfp*, *myo-2>mCherry*]; *qnEx310* [P*ida-1>flp-13*,P*rab-3>mCherry*]

The strain RB1837, containing *frpr-4*(*ok2376*), was obtained from the CGC. *frpr-4*(*ok2376*) was outcrossed to N2 three times to create the strain NQ385. The presence of the *ok2376* deletion was detected by PCR.

### Molecular biology, transgenics and integrations

We constructed DNA constructs using overlap-extension polymerase chain reaction (PCR), as previously described [34]. Oligonucleotides used are listed in S2 Table. **C**onstructs were made by amplifying sequences from genomic DNA, GFP from the Andy Fire vector pPD95.75, NLS:gfp from the Andy Fire vector pPD122.13, and mCherry from pCFJ90 (Addgene).

Transgenic animals were created by microinjection [35] using a Leica DMIRB inverted DIC microscope equipped with an Eppendorf Femtojet microinjection system. Either the wild-type strain N2 or the *unc-119* mutant strain EG4322 animals were injected with 2–50 ng/μl of each construct in combination with one of the following injection markers: 5ng/μl pCFJ90 (P*myo-2>mCherry*), or 5ng/μl pPD118.33 (*Pmyo-2*>*gfp*). The DNA mix was adjusted to a final concentration of 150 ng/μl by adding 1 kb DNA ladder (New England Biolabs) or the plasmid pCFJ151 (*unc-119*(+)). The fosmid WRM0630bE03 was injected into *frpr-4*(*ok2376*) animals at a concentration of 2 ng/μl. For behavioral experiments using transgenic animals carrying extrachromosomal arrays, at least two lines were analyzed. The integrated transgene *qnIs195* was constructed as previously described [36] by UV irradiation of strains carrying the extrachromosomal transgenes *qnEx195* [36] and then out-crossed to the wild-type strain four times before analysis.

### Microscopy and fluorescence

For GFP and differential interference contrast imaging, animals were mounted on 5% agar pads, immobilized with 15mM levamisole and observed through a 63X or 100X oil-immersion objective lens on a Leica DM5500B microscope. Leica LAS software was used to capture and analyze images.

### RNA interference

A 3.6 kb genomic fragment spanning a portion of the *frpr-4* gene (C54A12.2) (See S2 Fig) was amplified from genomic DNA using the primers oNQ627 and oNQ628 (S1 Table), which contained T7 5’ tails. The PCR product was purified (QIAquick PCR Purification Kit, Qiagen) and used in an *in vitro* T7 RNA polymerase transcription kit (New England Biolabs) to produce double stranded RNA. Double stranded RNA was injected into the intestine or gonad of wild-type, first-day old adults at a concentration of 200–300 ng/μl. The progeny of the injected worms were analyzed for their ability to become quiescent in response to heat stress.

### Feeding quiescence after heat exposure

On the day prior to the experiments, 15–25 L4 animals were transferred to Petri dishes containing 12 mL of 1.7% NGM agar seeded with DA837 bacteria. Two different experimental approaches were used to heat stress first day adult animals. Protocol 1 (mild heat shock): On the day of the experiment, the plates housing the worms were wrapped in parafilm and submerged in a 35°C water bath for 30 minutes. During a single experiment, the various genotypes were staggered with regards to the time that they entered and exited the water bath. After removing the plates from the water bath, the worms were observed at room temperature (21–23°C) every 10 minutes for 60 minutes for the presence of pharyngeal pumping using a Leica MZ16 stereomicroscope at total magnifications of at least 50X. Protocol 2: In experiments assessing the effect of exposure temperature on feeding quiescence, animals were subjected to precisely 30 minutes of heat at a specific temperature. NGM agar plates seeded with OP50 were pre-heated to the desired temperature for 20 minutes by submerging them in a water bath. Room temperature (21–23°C) first day adults were transferred to the pre-heated plates and immediately submerged in a water bath set at the same temperature for 30 minutes. Immediately after the heat exposure, the worms were transferred to room temperature plates seeded with bacteria and then assessed every 10 minutes for 60 minutes for the presence of pharyngeal pumping. In all cases, Statistical comparisons were made between genotypes tested simultaneously. Experiments were performed by investigators blinded to the genotype of the animals.

### Locomotion quiescence after heat exposure

To measure locomotion quiescence, we monitored first day adult worms cultivated on an agar surface in concave polydimethylsiloxane (PDMS) wells seeded with DA837 bacteria [22, 24] following heat exposure. First, NGM plates were preheated in a water bath for 20 minutes to the desired heat exposure temperature. Animals grown on room temperature plates were transferred to the pre-warmed plates and heat shocked for 30 minutes. Following heat exposure, single worms were transferred to the agar surface within a PDMS well. The microchip loaded with the worms was placed in a 50 ml petri dish, along with a wet Kimwipe, to prevent desiccation, and the plate was then sealed with Parafilm. Using a USB 2.0 Monochrome Industrial Camera (The Imaging Source^®^), dark-field images were taken every ten seconds for 90 minutes. Images were analyzed with the frame subtraction algorithm [21, 37, 38] using custom MATLAB software.

### Locomotion Quiescence analysis for *frpr-4* overexpression

We placed one first-day old adult in each of two adjacent concave polydimethylsiloxane (PDMS) wells filled with NGM agar and seeded with DA837 bacteria. In each experiment, we placed one experimental animal into one well and one control animal into an adjacent well. The PDMS was then placed on a Diagnostics Instruments microscope base and illuminated for bright-field microscopy, using white light supplied to the base with a fiber optic cable from a Schott DCR III light source. A camera (659 × 494 pixels, scA640-70fm, Basler Vision Technologies) mounted on a Zeiss Stemi 2000 stereomicroscope captured an image of both wells every 10 seconds with an 8-bit grayscale resolution. At this magnification and camera acquisition setting, the spatial resolution was 12.5 micrometers^2^ per pixel. We monitored animals for one hour beginning 15 minutes after transfer to the PDMS wells and used a machine vision frame subtraction principle to identify 10-second epochs of behavioral quiescence [21, 37, 38].

### Quantitative PCR

First-day adult animals were collected prior to heat shock and then at seven additional time intervals (0, 15, 30, 45, 60, 120, and 180 minutes) following a 37°C heat shock for 30 minutes. Total RNA was collected from each group of worms using an RNAeasy mini kit (Qiagen), and cDNA was synthesized using the SuperScript one-step RT-PCR system (Invitrogen). We performed three or more biological replicates, each of which was performed using several 100 first day adult animals, during each collection time point, and for each biological replicate we calculated the average of two technical replicates. Real-time PCR was performed using Taqman Gene Expression Mastermix on an Applied Biosystems 7500 platform at the core services within the Penn Center for AIDS Research, an NIH-funded program (P30 AI 045008). Oligonucleotides used are listed in S2 Table. Relative mRNA concentrations were determined by the delta-delta method [39] by normalization to the expression of the gene *pmp-3*, which has been shown to show little expression variance [40].

### Identification of *frpr-4* gene structure

To determine the 3’ end of *frpr-4* and identify potentially different isoforms, we used a 3’RACE method [14]. We collected wild-type worms of mixed stages from 5–10 plates in which the bacteria had recently depleted, isolated total RNA using an RNAeasy mini kit (Qiagen), and generated a cDNA library using SuperScript one-step RT-PCR system (Invitrogen). We used the primer oNQ549 (Identical to Q_T_, as described by Scotto-Lavino et al [14]) to generate the cDNA library. We then used the FRPR-4 gene-specific primer oNQ578 together with oNQ550(Identical to Q_o_ [14]) in an initial round of PCR to amplify a portion of the *frpr-4* cDNA. This PCR product was then used as the template in a PCR reaction with the nested primers oNQ579 and oNQ551(Identical to Q_i_ [14]) to amplify the final cDNA ends. Finally, we cloned the cDNAs into a pCR2.1-TOPO Vector (Invitrogen) and sequenced the cloned inserts.

### Receptor ligand interactions *in cellulo*

Total RNA was collected using an RNAeasy mini kit (Qiagen) from wild-type animals harvested from mixed stage populations grown on five NGM plates. cDNA was synthesized using the SuperScript one-step RT-PCR system (Invitrogen). FRPR-4A cDNA was PCR amplified, directionally cloned into the pcDNA3.1(+) TOPO expression vector (LifeTechnologies), and sequenced to confirm that no errors were introduced during the PCR or cloning steps.

Receptor activation was studied in Chinese hamster ovary cells (CHO) stably expressing apo-aequorin (mtAEQ) targeted to the mitochondria as well as the human G_alpha16_ subunit. The CHO/mtAEQ/G_alpha16_ cells were cultured in Ham’s F12 medium (Sigma), containing 10% fetal bovine serum (FBS), 100 UI/ml of penicillin/streptomycin, 250 μg/ml Zeocin and 2.5 μg/ml Fungizone (Amphoterin B). Cell lines were grown at 37°C in a humidified atmosphere of 5% CO_2_ and were diluted fifteen-fold every third day. CHO/mtAEQ/G_alpha16_ cells were transiently transfected with the FRPR-4 cDNA construct or the empty pcDNA3.1(+) vector using the FuGENE 6 transfection reagent (Promega), according to the manufacturer’s instructions. Cells expressing the receptor were collected 2 days post-transfection in BSA medium (DMEM/HAM’s F12 with 15 mM HEPES, without phenol red, supplemented with 0.1% BSA) and loaded with 5 μM coelenterazine h (Invitrogen) for 4 hours to reconstitute the holo-enzyme aequorin. The cells were plated at a density of 25,000 cells/well and exposed to synthetic peptides at a concentration of 10μM in BSA medium. Aequorin bioluminescence was recorded for 30 seconds on a Mithras LB 940 luminometer (Berthold Technologies) in quadruplicate. For dose-response evaluations, after 30 seconds of ligand-stimulated calcium measurements, Triton X-100 (0.1%) was added to the well to obtain a measure of the maximum cell Ca^2+^ response. BSA medium without the peptides was used as a negative control and 1 μM ATP was used to check the functional response of the cells. Cells transfected with the pcDNA3.1 empty vector were used as a negative control for the effect of the receptor. EC_50_ values were calculated from dose-response curves, constructed using a computerized nonlinear regression analysis, with a sigmoidal dose-response equation (Sigmaplot 9.0).

### Measuring body bend angle

Wild-type, *frpr-4* (*ok2376*), *flp-13*(*tm2427*), *frpr-4*(OE), *flp-13*(*tm2427*); *frpr-4*(OE), DVA>*frpr-4*(OE), *flp-13*(-);DVA>*frpr-4*(OE) and *flp-13*(*-*); DVA:*frpr-4*(OE); ALA:*flp-13* first-day adult worms were transferred to either fully seeded plates or unseeded plates. Worms were video monitored for at least 2 minutes using a camera (USB 2.0 Monochrome Industrial camera, ImageSource) mounted on a Leica Microsystems MZ10F stereomicroscope (see S3–S7 Movies). Videos were analyzed using ImageJ software [41]. Videos of 10 or more individual animals for each genotype were captured. For each individual worm, we drew lines from the peak of one bend to the peak of the opposite bend (See Fig 3). We did this for a minimum of 3 body bends for each individual and averaged the bend angles measured for that individual. We then calculated the average and standard error of the mean of all individuals within a given genotype.

### Phylogenetic Tree construction

We first aligned *C*. *elegans* [5] and *Drosophila melanogaster* [42] predicted neuropeptide receptors using ClustalW version 2.0 [43]. The alignment was then used as a template to construct a maximum-likelihood tree using the MEGA6 software [44].

## Supporting Information

S1 Fig A phylogenetics tree consisting of all *D*. *melanogaster* and *C*. *elegans* predicted neuropeptide receptors, as listed in references [5, 42]. FRPR-4 is most closely related to *D*. *melanogaster* FR.
(TIF)

S2 Fig The gene model and isoforms of the *frpr-4* gene predicted by wormbase.org (top model) and experimentally determined (bottom three models) by 3’RACE. Arrows denote inverted repeats flanking intronic DNA, which separates the two parts of the 3’UTR (gray) in isoform A and separates the last two coding exons in isoforms B and C. The *ok2376* deletion removes the first two exons of all three isoforms. It also removes 300 nucleotides of upstream regulatory DNA. The location of *gfp* in the strains NQ408, NQ459 and NQ460 and the location of the DNA used as template for generating double stranded RNA in the RNAi experiments are marked at the bottom of the figure.
(TIF)

S3 Fig Alignment of *C*.*elegans* FRPR-4 with *C*. *briggsae* FRPR-4 supports the gene structure determined by cDNA sequencing. Blue denote the most 5’ experimentally-determined coding exon. Red denotes the sixth coding exon of FRPR-4A and FRPR-4B, which is absent in FRPR-4C (not shown) and in *C*. *briggsae*. Green denotes the most 3’ coding exon, which is present in FRPR-4A and in *C*. *briggsae*. Grey denotes the 3’RACE validated 3’-untranslated region (3’UTR). *C*. *briggsae* does not possess the retrotranspon that was observed in the 3’ UTR of the *C*. *elegans frpr-4* gene.
(TIF)

S4 Fig Alignment of (I/V)RF neuropeptides that activate and do not activate FRPR-4A. Hydrophobic amino acids are yellow, charged amino acids are red, and polar but uncharged amino acids are blue. On average the peptides that do not activate are shorter (mean±SD = 7.9±1.3 amino acids) than those that activate (9.4±1.4 amino acids; p = 0.002). No other feature is consistently different between the two groups.
(TIF)

S5 Fig Transgenic lines that over express either *frpr-4* or *frpr-4*:*gfp* induce behavioral quiescence. A significant fraction of first-day old adult transgenic animals carrying additional copies of the *frpr-4* gene (middle bar) or additional copies of *frpr-4*:*gfp* translational reporters (right bar) are quiescent. Shown is the average ± s.e.m of three trials using two independent transgenic lines of each genotype, with each trial containing 20–30 animals of each genotype. (Students t-test, ***P < .001).
(TIF)

S6 Fig *frpr-4* mRNA is not induced by heat stress. (TIF)

S7 Fig FLP-13 peptides are not required for *frpr-4*-induced quiescence and *frpr-4* is not required for *flp-13*-induced quiescence. Machine vision analysis shows that the *flp-13*(*tm2427*) mutation does not significantly suppress the elevated total quiescence (**A**), quiescence bout frequency (**B**), or quiescence bout duration (**B**) induced by *frpr-4* overexpression. (**C**) Direct observation shows that the *frpr-4*(*ok2376*) mutation does not suppress the elevated quiescence induced by *flp-13* overexpression. Shown is the average ± s.e.m fraction of animals quiescent for feeding and locomotion two hours after heat-shock promoter induced expression of *flp-13*. Shown in A and B is the average of >15 trials with 1 animal of each genotype per trial. Shown in C is the average of 2 trials with >25 animals per trial.
(TIF)

S8 Fig *frpr-4* has a broad expression pattern. (**A**) Transgenic animals carrying an *frpr-4*:*gfp* translational reporter show GFP localization to the membrane of multiple neurons, including the RIA neurons (identified using the *Pglr-3>mCherry* marker) and PVM neuron (identified based on location and morphology), as well as body muscle. (**B**) Transgenic animals expressing a P*frpr-4*>NLS:*gfp* transcriptional reporter shows additional expression in the AVE neuron (which co-expresses the gene *opt-3*, marked in red in the left panel in B), the I1 pharyngeal neuron (identified based on location and morphology) and other head neurons. In the left panel in A and in the right panel in B, the RIA neurons co-express the gene *glr-3*, which is marked in red.
(TIF)

S1 Movie Several first-day old adult wild-type animals (on the left) and first-day old adult transgenic animals expressing an integrated multi-copy array of the gene *frpr-4* (strain NQ480, on the right). Whereas the wild-type animals are continuously active, *frpr-4* over-expressing animals have spontaneous bouts of locomotion quiescence. The movie is played at 16 times the real speed.
(MP4)

S2 Movie Two first-day old adult animals over-expressing *frpr-4* (strain NQ480). The left worm is foraging whereas the right worm is quiescent. In response to two dish taps, both worms move, one forward and the other backwards. The movie is played at real speed.
(MP4)

S3 Movie One first day adult N2 (wild-type) animal moving on an unseeded agar surface. The movie is played at 16 times the real speed.
(MP4)

S4 Movie One first day adult NQ480 (*frpr-4*(OE)) animal moving on an unseeded agar surface. Body bends are deeper than those of N2 animals (reflecting smaller bend angles of body bends by the transgenic animals). The movie is played at 16 times the real speed.
(MP4)

S5 Movie A first day adult NQ588 (*flp-13*(-); *frpr-4*(OE)) animal moving on an unseeded plate. Body bends appear similar to those of N2 animals. The movie is played at 16 times the real speed.
(MP4)

S6 Movie A first day adult NQ756 (DVA>*frpr-4*(OE)) animal moving on an unseeded plate. Body bends are deeper than those of N2 animals. The move is played at 8 times the real speed.
(MP4)

S7 Movie A first day adult NQ756 (*flp-13*(-); DVA>*frpr-4*(OE)) animal moving on an unseeded plate. Body bends appear similar to those of N2 animals. The movie is played at 8 times the real speed.
(MP4)

S8 Movie A first day adult SJU47 (*flp-13*(-); DVA>*frpr-4*(OE); ALA>*flp-13*) animal moving on an unseeded plate. Body bends are deeper than those of N2 animals. The movie is played at 8 times the real speed.
(MP4)

S1 Table Sequences of the cDNA of the experimentally verified FRPR-4 isoforms and the presumed transposable element located in the most 3’ intron of *frpr-4*. (DOCX)

S2 Table Oligonucleotides used in this study. Most DNA construct was made using overlap-extension PCR, as previously described. **£**The FRPR-4A cDNA was amplified from a *C*.*elegans* cDNA library (See Materials and Methods). **§**The PCR product used to make dsRNA (See Materials and Methods) was amplified from genomic DNA with PCR-engineered tails containing T7 promoters.
(DOCX)
