# Supplementary figures and images for: FRPR-4 Is a G-Protein Coupled Neuropeptide Receptor That Regulates Behavioral Quiescence and Posture in Caenorhabditis elegans
Source: PLoS One. 2015 Nov 16;10(11):e0142938. doi: 10.1371/journal.pone.0142938 (PMC4646455; doi:10.1371/journal.pone.0142938)

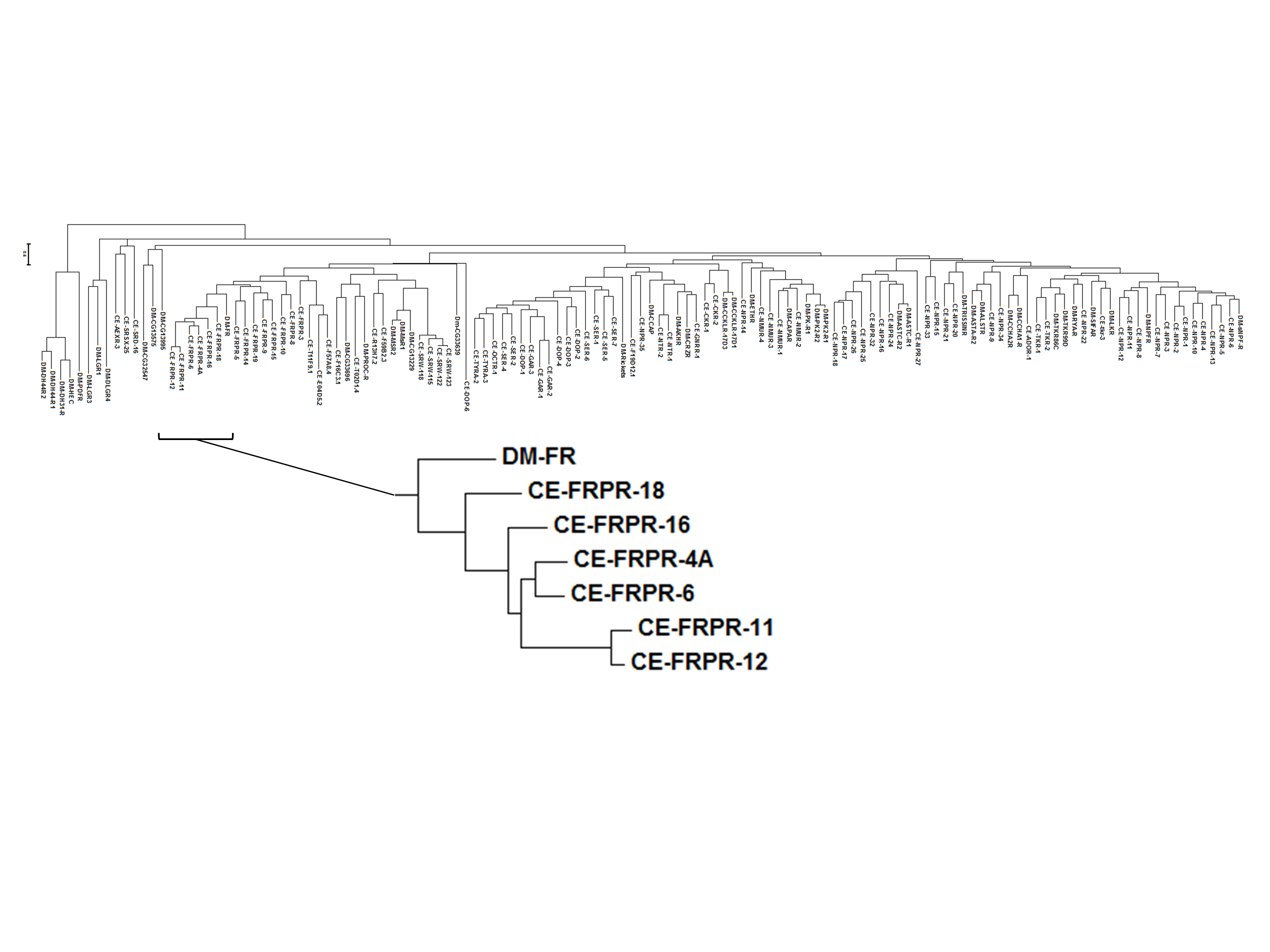

Supplement: S1 Fig — FRPR-4 is most closely related to D. melanogaster FR. (TIF) [file pone.0142938.s001.TIF]

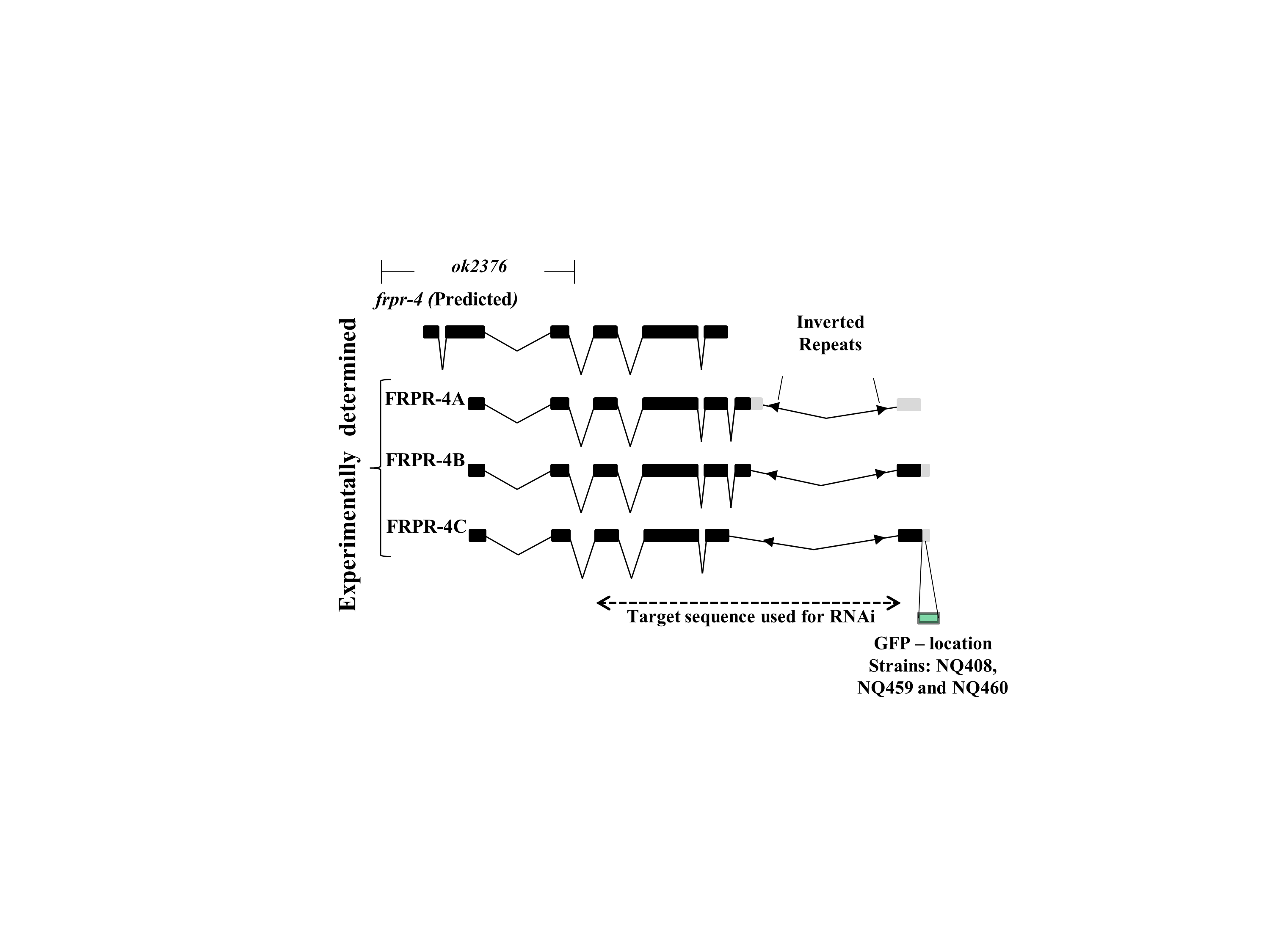

Supplement: S2 Fig — Arrows denote inverted repeats flanking intronic DNA, which separates the two parts of the 3’UTR (gray) in isoform A and separates the last two coding exons in isoforms B and C. The ok2376 deletion removes the first two exons of all three isoforms. It also removes 300 nucleotides of upstream regulatory DNA. The location of gfp in the strains NQ408, NQ459 and NQ460 and the location of the DNA used as template for generating double stranded RNA in the RNAi experiments are marked at the bottom of the figure. (TIF) [file pone.0142938.s002.TIF]

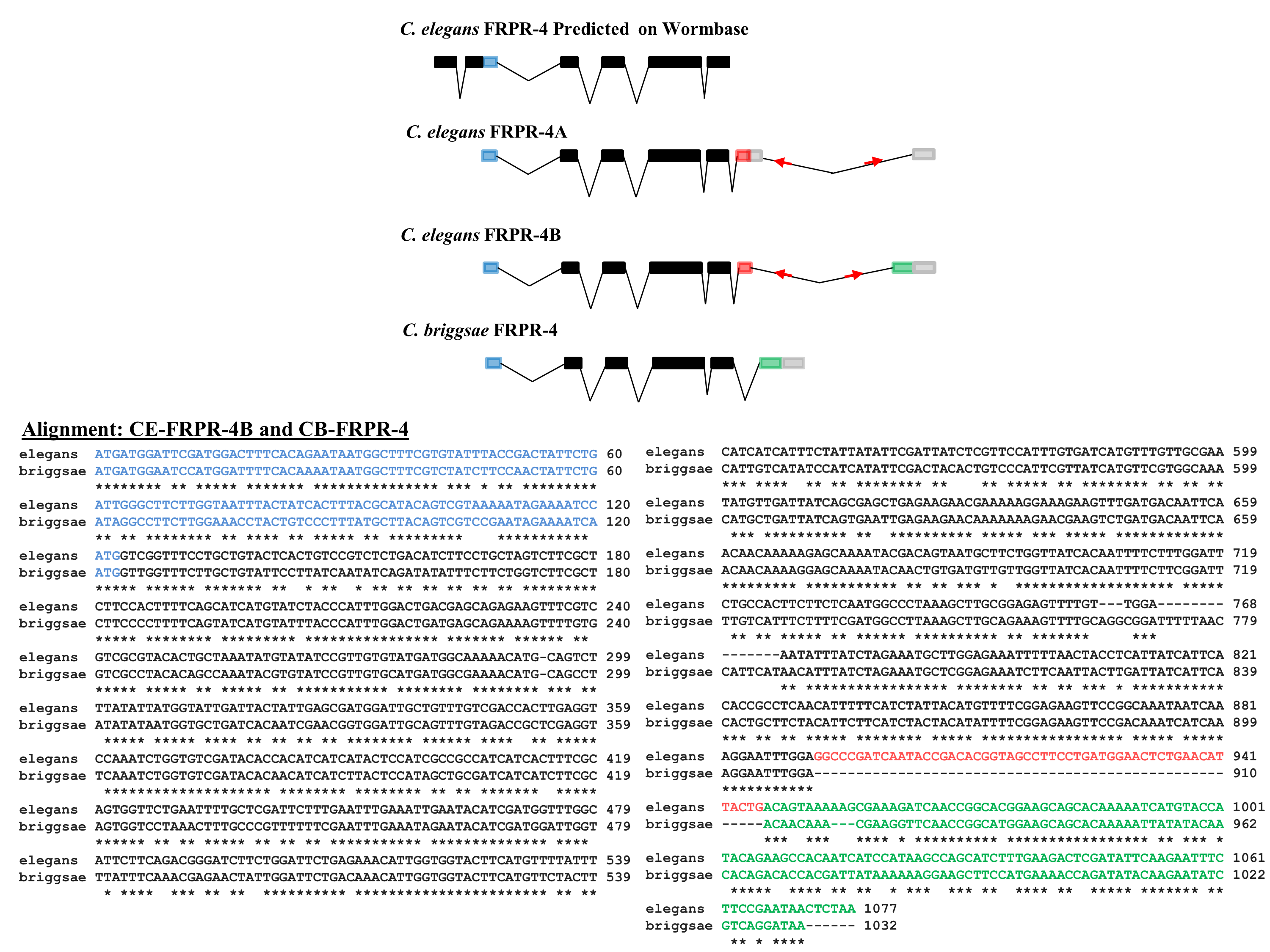

Supplement: S3 Fig — Blue denote the most 5’ experimentally-determined coding exon. Red denotes the sixth coding exon of FRPR-4A and FRPR-4B, which is absent in FRPR-4C (not shown) and in C. briggsae. Green denotes the most 3’ coding exon, which is present in FRPR-4A and in C. briggsae. Grey denotes the 3’RACE validated 3’-untranslated region (3’UTR). C. briggsae does not possess the retrotranspon that was observed in the 3’ UTR of the C. elegans frpr-4 gene. (TIF) [file pone.0142938.s003.TIF]

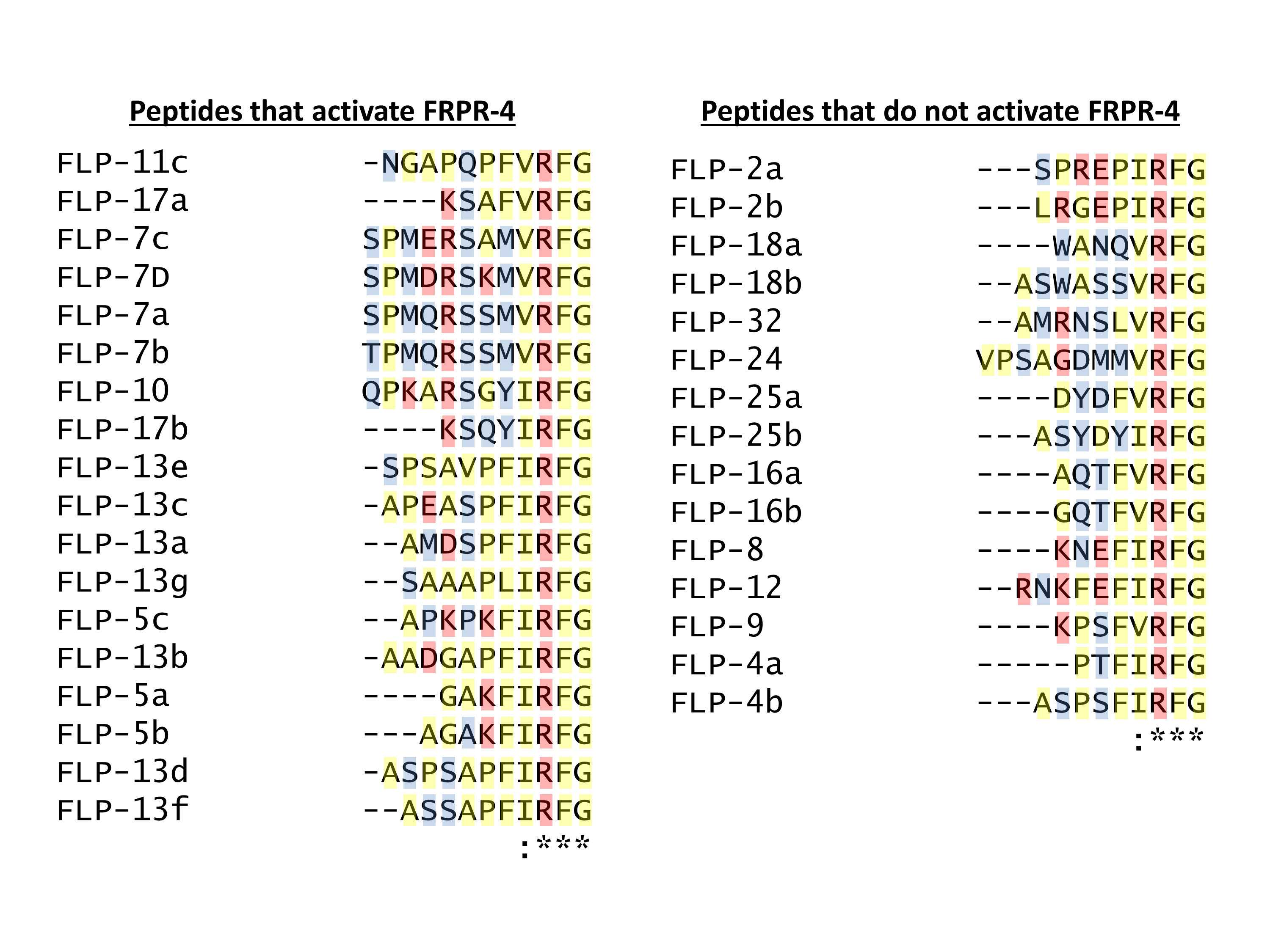

Supplement: S4 Fig — Hydrophobic amino acids are yellow, charged amino acids are red, and polar but uncharged amino acids are blue. On average the peptides that do not activate are shorter (mean±SD = 7.9±1.3 amino acids) than those that activate (9.4±1.4 amino acids; p = 0.002). No other feature is consistently different between the two groups. (TIF) [file pone.0142938.s004.TIF]

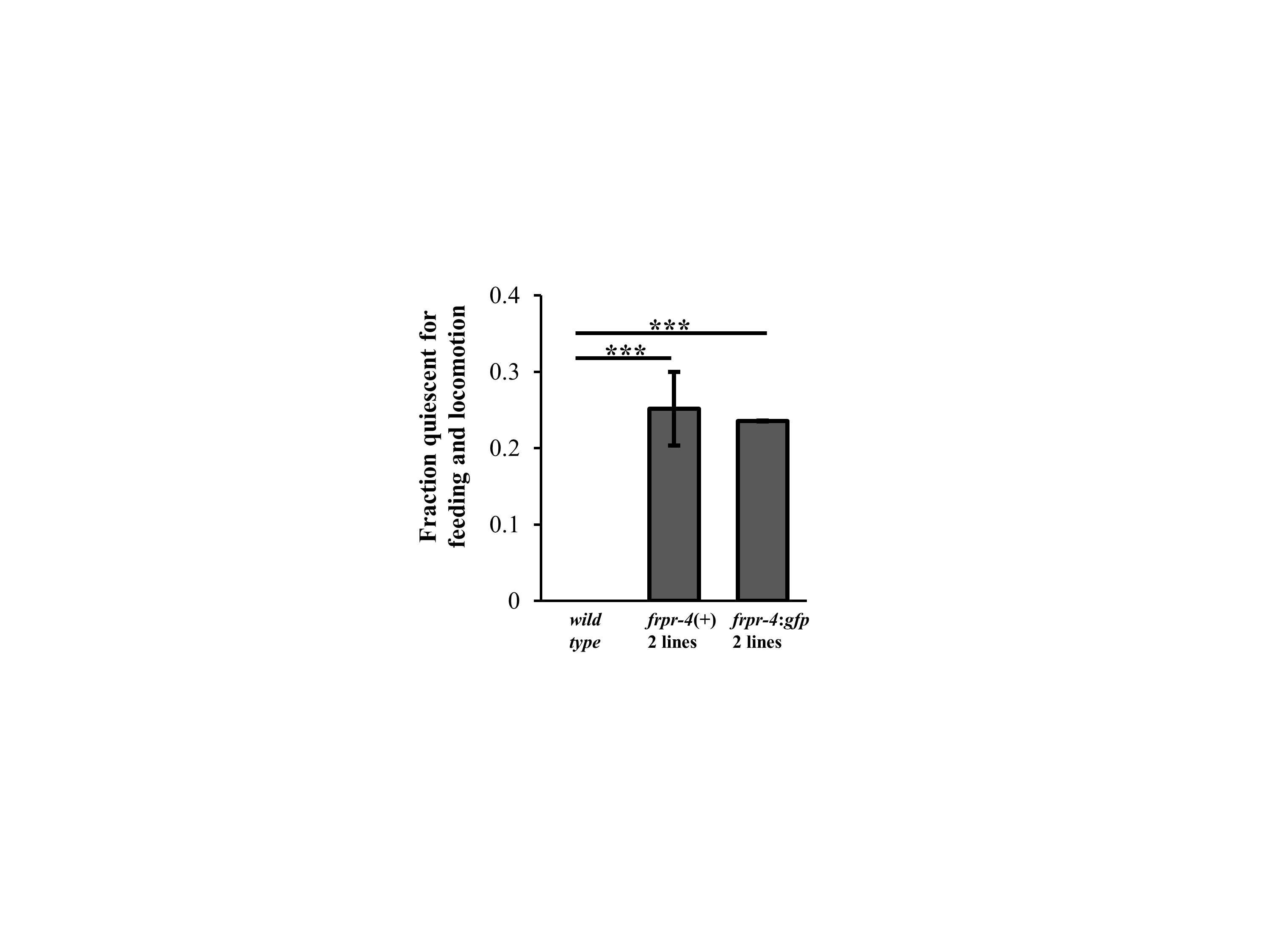

Supplement: S5 Fig — A significant fraction of first-day old adult transgenic animals carrying additional copies of the frpr-4 gene (middle bar) or additional copies of frpr-4:gfp translational reporters (right bar) are quiescent. Shown is the average ± s.e.m of three trials using two independent transgenic lines of each genotype, with each trial containing 20–30 animals of each genotype. (Students t-test, ***P < .001). (TIF) [file pone.0142938.s005.TIF]

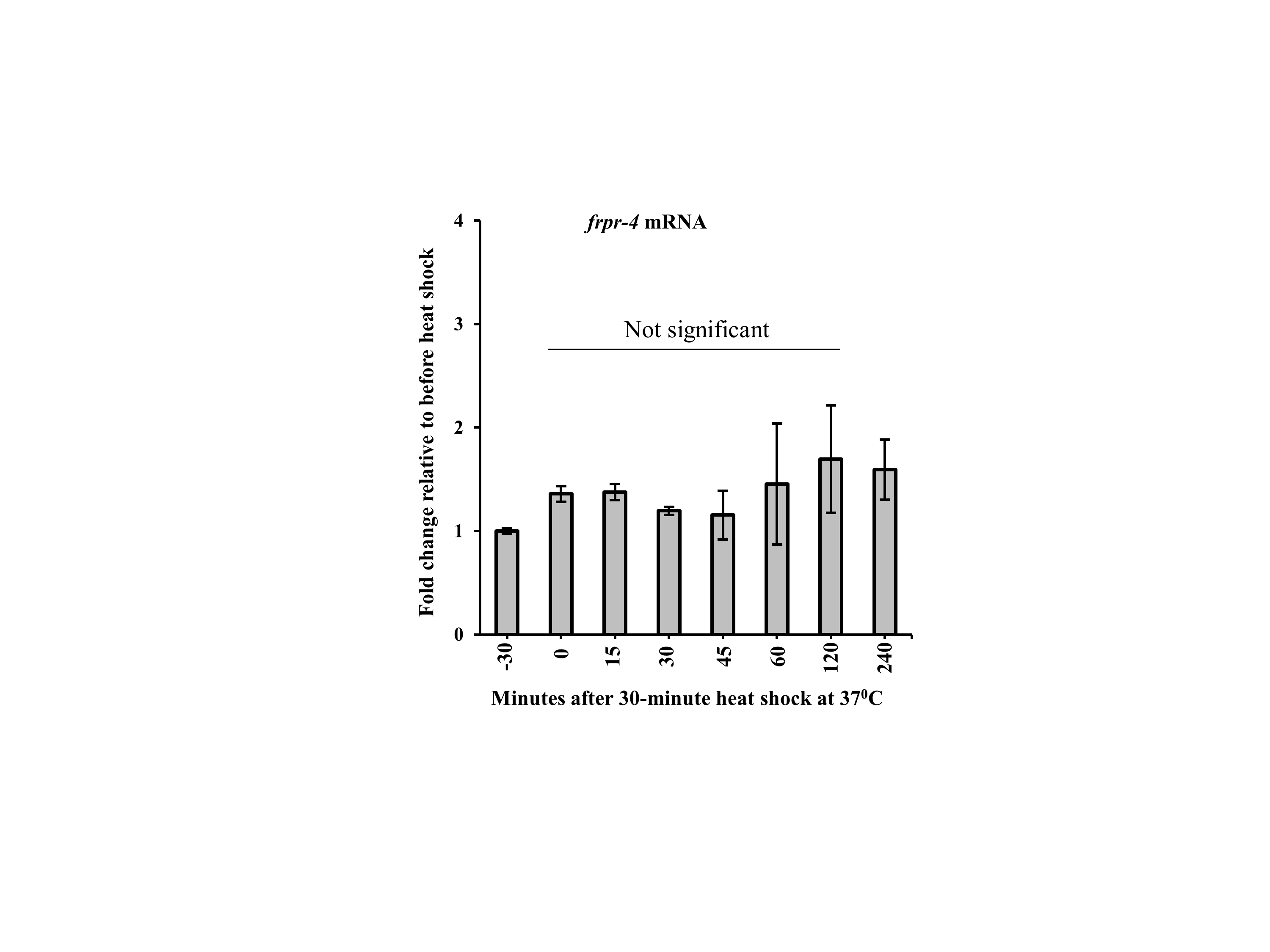

Supplement: S6 Fig — (TIF) [file pone.0142938.s006.TIF]

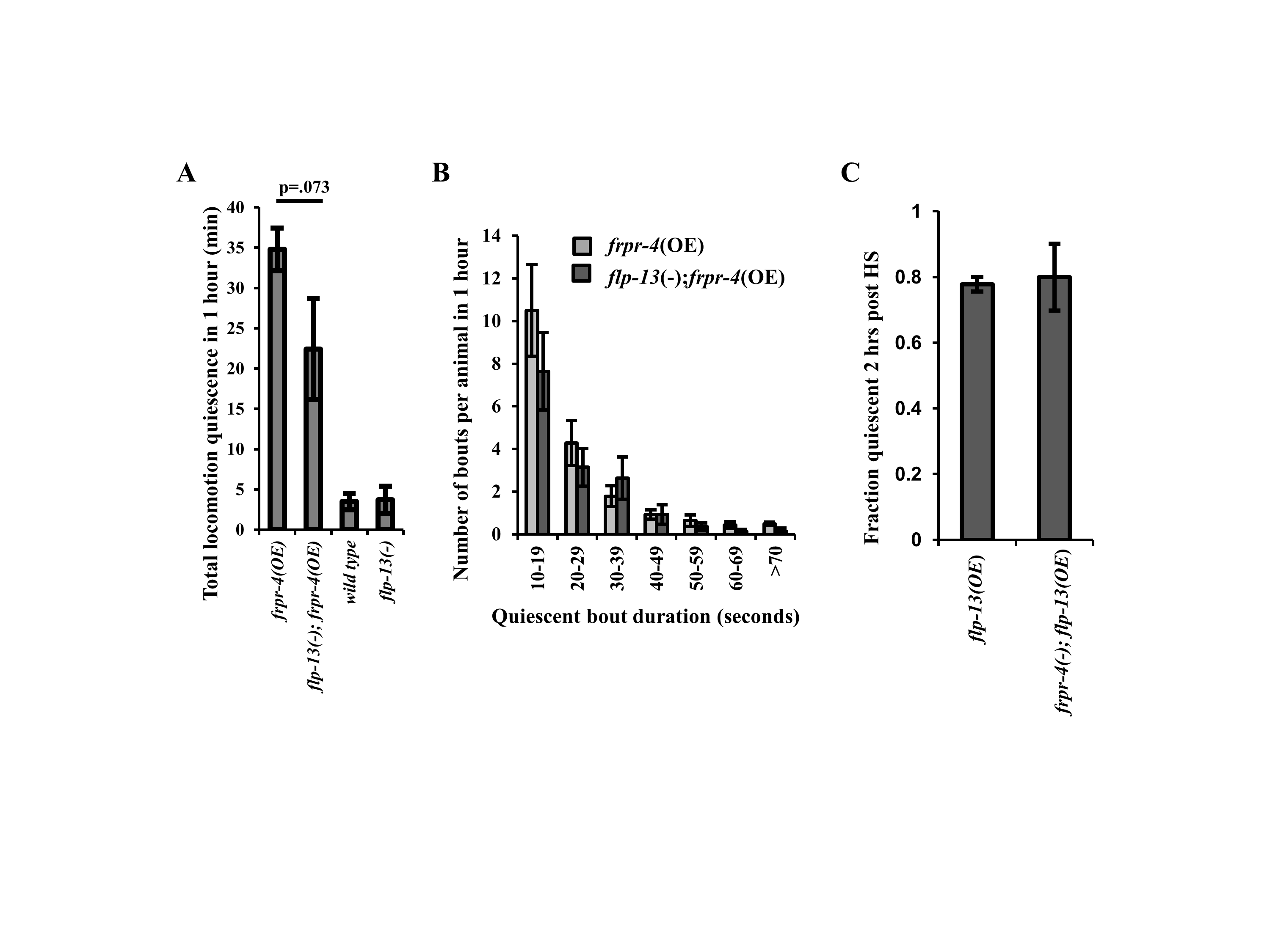

Supplement: S7 Fig — Machine vision analysis shows that the flp-13(tm2427) mutation does not significantly suppress the elevated total quiescence (A), quiescence bout frequency (B), or quiescence bout duration (B) induced by frpr-4 overexpression. (C) Direct observation shows that the frpr-4(ok2376) mutation does not suppress the elevated quiescence induced by flp-13 overexpression. Shown is the average ± s.e.m fraction of animals quiescent for feeding and locomotion two hours after heat-shock promoter induced expression of flp-13. Shown in A and B is the average of >15 trials with 1 animal of each genotype per trial. Shown in C is the average of 2 trials with >25 animals per trial. (TIF) [file pone.0142938.s007.TIF]

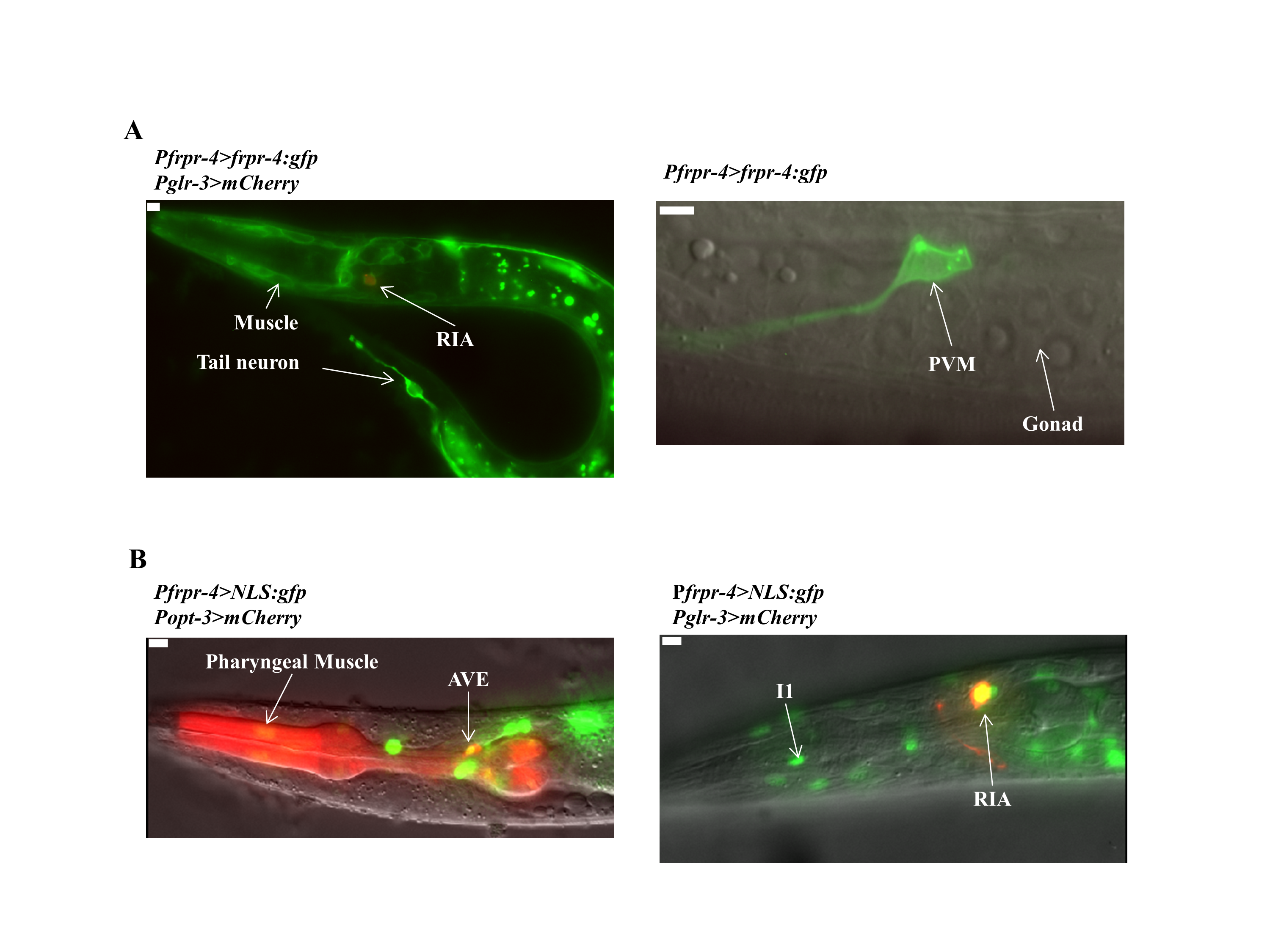

Supplement: S8 Fig — (A) Transgenic animals carrying an frpr-4:gfp translational reporter show GFP localization to the membrane of multiple neurons, including the RIA neurons (identified using the Pglr-3>mCherry marker) and PVM neuron (identified based on location and morphology), as well as body muscle. (B) Transgenic animals expressing a Pfrpr-4>NLS:gfp transcriptional reporter shows additional expression in the AVE neuron (which co-expresses the gene opt-3, marked in red in the left panel in B), the I1 pharyngeal neuron (identified based on location and morphology) and other head neurons. In the left panel in A and in the right panel in B, the RIA neurons co-express the gene glr-3, which is marked in red. (TIF) [file pone.0142938.s008.TIF]
