# Supplementary material for: FRPR-4 Is a G-Protein Coupled Neuropeptide Receptor That Regulates Behavioral Quiescence and Posture in Caenorhabditis elegans
Source: PLoS One. 2015 Nov 16;10(11):e0142938. doi: 10.1371/journal.pone.0142938 (PMC4646455; doi:10.1371/journal.pone.0142938)
Supplement: S1 Table — (DOCX) [file pone.0142938.s017.docx]

| **FRPR-4A cDNA** |
| --- |
| ATGATGGATTCGATGGACTTTCACAGAATAATGGCTTTCGTGTATTTACCGACTATTCTGATTGGGCTTCTTGGTAATTTACTATCACTTTACGCATACAGTCGTAAAAATAGAAAATCCATGGTCGGTTTCCTGCTGTACTCACTGTCCGTCTCTGACATCTTCCTGCTAGTCTTCGCTCTTCCACTTTTCAGCATCATGTATCTACCCATTTGGACTGACGAGCAGAGAAGTTTCGTCGTCGCGTACACTGCTAAATATGTATATCCGTTGTGTATGATGGCAAAAACATGCAGTCTTTATATTATGGTATTGATTACTATTGAGCGATGGATTGCTGTTTGTCGACCACTTGAGGTCCAAATCTGGTGTCGATACACCACATCATCATACTCCATCGCCGCCATCATCACTTTCGCAGTGGTTCTGAATTTTGCTCGATTCTTTGAATTTGAAATTGAATACATCGATGGTTTGGCATTCTTCAGACGGGATCTTCTGGATTCTGAGAAACATTGGTGGTACTTCATGTTTTATTTCATCATCATTTCTATTATATTCGATTATCTCGTTCCATTTGTGATCATGTTTGTTGCGAATATGTTGATTATCAGCGAGCTGAGAAGAACGAAAAAGGAAAGAAGTTTGATGACAATTCAACAACAAAAAGAGCAAAATACGACAGTAATGCTTCTGGTTATCACAATTTTCTTTGGATTCTGCCACTTCTTCTCAATGGCCCTAAAGCTTGCGGAGAGTTTTGTTGGAAATATTTATCTAGAAATGCTTGGAGAAATTTTTAACTACCTCATTATCATTCACACCGCCTCAACATTTTTCATCTATTACATGTTTTCGGAGAAGTTCCGGCAAATAATCAAAGGAATTTGGAGGCCCGATCAATACCGACACGGTAGCCTTCCTGATGGAACTCTGAACATTACTGACAGGTATCAAAAAATTTTGTGA |
| **FRPR-4B cDNA** |
| ATGATGGATTCGATGGACTTTCACAGAATAATGGCTTTCGTGTATTTACCGACTATTCTGATTGGGCTTCTTGGTAATTTACTATCACTTTACGCATACAGTCGTAAAAATAGAAAATCCATGGTCGGTTTCCTGCTGTACTCACTGTCCGTCTCTGACATCTTCCTGCTAGTCTTCGCTCTTCCACTTTTCAGCATCATGTATCTACCCATTTGGACTGACGAGCAGAGAAGTTTCGTCGTCGCGTACACTGCTAAATATGTATATCCGTTGTGTATGATGGCAAAAACATGCAGTCTTTATATTATGGTATTGATTACTATTGAGCGATGGATTGCTGTTTGTCGACCACTTGAGGTCCAAATCTGGTGTCGATACACCACATCATCATACTCCATCGCCGCCATCATCACTTTCGCAGTGGTTCTGAATTTTGCTCGATTCTTTGAATTTGAAATTGAATACATCGATGGTTTGGCATTCTTCAGACGGGATCTTCTGGATTCTGAGAAACATTGGTGGTACTTCATGTTTTATTTCATCATCATTTCTATTATATTCGATTATCTCGTTCCATTTGTGATCATGTTTGTTGCGAATATGTTGATTATCAGCGAGCTGAGAAGAACGAAAAAGGAAAGAAGTTTGATGACAATTCAACAACAAAAAGAGCAAAATACGACAGTAATGCTTCTGGTTATCACAATTTTCTTTGGATTCTGCCACTTCTTCTCAATGGCCCTAAAGCTTGCGGAGAGTTTTGTTGGAAATATTTATCTAGAAATGCTTGGAGAAATTTTTAACTACCTCATTATCATTCACACCGCCTCAACATTTTTCATCTATTACATGTTTTCGGAGAAGTTCCGGCAAATAATCAAAGGAATTTGGAGGCCCGATCAATACCGACACGGTAGCCTTCCTGATGGAACTCTGAACATTACTGACAGTAAAAAGCGAAAGATCAACCGGCACGGAAGCAGCACAAAAATCATGTACCATACAGAAGCCACAATCATCCATAAGCCAGCATCTTTGAAGACTCGATATTCAAGAATTTCTTCCGAATAACTCTAA |
| **FRPR-4C cDNA** |
| ATGATGGATTCGATGGACTTTCACAGAATAATGGCTTTCGTGTATTTACCGACTATTCTGATTGGGCTTCTTGGTAATTTACTATCACTTTACGCATACAGTCGTAAAAATAGAAAATCCATGGTCGGTTTCCTGCTGTACTCACTGTCCGTCTCTGACATCTTCCTGCTAGTCTTCGCTCTTCCACTTTTCAGCATCATGTATCTACCCATTTGGACTGACGAGCAGAGAAGTTTCGTCGTCGCGTACACTGCTAAATATGTATATCCGTTGTGTATGATGGCAAAAACATGCAGTCTTTATATTATGGTATTGATTACTATTGAGCGATGGATTGCTGTTTGTCGACCACTTGAGGTCCAAATCTGGTGTCGATACACCACATCATCATACTCCATCGCCGCCATCATCACTTTCGCAGTGGTTCTGAATTTTGCTCGATTCTTTGAATTTGAAATTGAATACATCGATGGTTTGGCATTCTTCAGACGGGATCTTCTGGATTCTGAGAAACATTGGTGGTACTTCATGTTTTATTTCATCATCATTTCTATTATATTCGATTATCTCGTTCCATTTGTGATCATGTTTGTTGCGAATATGTTGATTATCAGCGAGCTGAGAAGAACGAAAAAGGAAAGAAGTTTGATGACAATTCAACAACAAAAAGAGCAAAATACGACAGTAATGCTTCTGGTTATCACAATTTTCTTTGGATTCTGCCACTTCTTCTCAATGGCCCTAAAGCTTGCGGAGAGTTTTGTTGGAAATATTTATCTAGAAATGCTTGGAGAAATTTTTAACTACCTCATTATCATTCACACCGCCTCAACATTTTTCATCTATTACATGTTTTCGGAGAAGTTCCGGCAAATAATCAAAGGAATTTGGAATAAAAAGCGAAAGATCAACCGGCACGGAAGCAGCACAAAAATCATGTACCATACAGAAGCCACAATCATCCATAAGCCAGCATCTTTGAAGACTCGATATTCAAGAATTTCTTCCGAATAA |
| **Presumed Transposable element (Inverted Repeats are bolded)** |
| **tactcaggcaactcaaatctaatatgaaaatattaaatagcaatatttttgtagt**aaaaacgagaaatagtgcgtccatatacgattactaaaaatttcgaatagatttcaattttgtctcaaaagattacaatttgatctcaaaatattgcgaaagtgtgaaaatttctaggaagacttccgttttatctcaaaatattactaatttgtctcaaaagactgcgaactagtctccgaagattgagatttcatctccgaagattgagatttcgtctcagaatattgcgattttgtctcaaaatattgcgaatttgtctctaaatattaaaaatgcattcatcgactattctagaactgttttgtgataactcgaagaacttttgttgccattgaggggagttgaacttgtccgtcgcttgtcgttgagtctatgaaccattatcttacctgttgagccatcctgtacgtgtcgcatctagatatagttattgcttaagtaaatagttgatgggcgcacgtccttttcgttacctttgatgggaaatcagtgacttttcgcaaattttctaagtgtcagagcgaaattggataactttctttgaaagcgaatgttgaaaaacgatttatttgttatgcttttaaaaatgttagaattgcaatttttccgaatttttgtcaaagagagaataacattttattttttcaatatttattttcactaacaatttgattaaaatgatgagtatactgggaacatgcttgctgttgacaatattttatgatttcaattgaaatcagaaaatctcaaacgcatcacgcgtgaatgaatagttgtaagtctactttaatgtatttgaccaatcttcgtgattgtttgactgaatttataatatgaccggccaattatctagaaaaaattacatgagtggacagatttgtgaattctgtggctttctctgcaaaaactccttctacaatatgcaacgaaaaatcaaatatccgttaacagtgagatactccattatatagattttcatgtagctgtttttttactttgaaaagtcttttatcatttggaagaaaagagaatttagaacggaacagtaatcataattgttgtatatatatatcacgatcatgtgaagccgatggaaatttggaaatggtgttttgtaatctttatggactgcaaaaagattgcaaattaatcttagaatattacacatttttttgtaatatttttagatttcagaaagattgcgaaccaatctcaaaagattgctatgtcatctcaaaatattacgaatttttcgtaatattttcagattcagaaatattgcgaatcaacctcaaaatattgcgaaatattctcaaaagattgctcaatctaaaaagatttcgaataagtctcggaatattgcgattttatcttgaaatatcaccca**actacaaaaatgttgctatttaatattttcatattagatttgagttgcctgagta** |
