# Supplementary material for: FRPR-4 Is a G-Protein Coupled Neuropeptide Receptor That Regulates Behavioral Quiescence and Posture in Caenorhabditis elegans
Source: PLoS One. 2015 Nov 16;10(11):e0142938. doi: 10.1371/journal.pone.0142938 (PMC4646455; doi:10.1371/journal.pone.0142938)
Supplement: S2 Table — Most DNA construct was made using overlap-extension PCR, as previously described. £The FRPR-4A cDNA was amplified from a C.elegans cDNA library (See Materials and Methods). §The PCR product used to make dsRNA (See Materials and Methods) was amplified from genomic DNA with PCR-engineered tails containing T7 promoters. (DOCX) [file pone.0142938.s018.docx]

| **Oligo Name** | **Description** | **Sequence** |
| --- | --- | --- |
| **P*frpr-4*::*frpr-4*::*gfp*** | | |
| oNQ920  oNQ921  oNQ907  oNQ908  oNQ472  oNQ771 | Forward - Binds >5kb upstream of *frpr-4*  Nested to oNQ920  Reverse - Before the TAA of FRPR-4A. Has *gfp* tail  Forward - Start of *gfp* in pPD95.75. Has *frpr-4* tail  Reverse - *unc-54* 3’UTR in pPD95.75  Nested to oNQ472 | CTTTTTTCAATTTGCATCCACCCC  CAATTTGCATCCACCCCCACTT  CAACTCCAGTGAAAAGTTCTTCTCCTTTACTCATCAAAATTTTTTGATACCTGTCAG  ACAGGTATCAAAAAATTTTGATGAGTAAAGGAGAAGAACTTTT  AAAAGAAGCTAAAAAACAAAGAAATTA  AACAAAAATAGGGGGTGGGAG |
| **P*frpr-4*:NLS:*gfp*** | | |
| oNQ920  oNQ921  oNQ922  oNQ923  oNQ924  oNQ925 | See Above  See Above  Reverse - Start of *frpr-4.* Adds NLS:*gfp* tail  Forward - Start of NLS:*gfp* in pPD122.13. Has *frpr-4* tail  Reverse - *unc-54* 3’UTR in pPD122.13  Nested to oNQ924 | CTTACGCTTCTTCTTTGGAGCAGTCATAAAGTCCATCGAATCCATCAT  ATGACTGCTCCAAAGAAGAAGC  ATTGTGTTCAGATGAGAGGAGC  GTTCAGATGAGAGGAGCGGAA |
| **P*opt-3*:mCherry** | | |
| oNQ1202  oNQ1203  oNQ1204  oQN1205  oNQ472  oNQ771 | Forward - >3kb upstream of *opt-3*  Nested to oNQ1202  Reverse - Start of *opt-3*. Has mCherry tail  Forward - Start of mCherry in pCFJ90*.* Has *opt-3* tail  See Above  See Above | GTAATTCAAATCCTGAAAATTCAAG  GAAAATTCAAGAACTATCCTACAGC  TGTGAAAGTCCATCGAATCCATGGTTAATTGAGATTCCTTTTGCTCC  GCTTTTTTGTACAAACTTGTCATGGTTAATTGAGATTCCTTTTGCTCC |
| **P*glr-3*:*mCherry*** | | |
| oNQ615  oNQ616  oNQ856  oNQ857  oNQ472  oNQ771 | Forward - >5kb upstream of *glr-3*  Nested to oNQ615  Reverse - Start of *glr-3*. Has mCherry tail  For - Start of mCherry from pCFJ90. Has *glr-3* tail  See Above  See Above | CTTCAATCTTCAAAAAAGGGCATT  CTTCAAAAAAGGGCATTAAAAACAGT  GAAAAGTTCTTCTCCTTTACTCATAATCGCAATCGACTTTTTCATGAT  CTTTTTTGTACAAACTTGTCATGAACATATGTTAATAGCAAATATT |
| **Genomic fragment for OE of *frpr-4*** | | |
| oNQ920  oNQ581 | See Above  Reverse - Binds +5605 to *frpr-4* start codon? | TGGTTAAAAGTCAAAACGGTTAAAAAGA |
| **P*twk-16*:*frpr-4*** | | |
| oNQ1377  oNQ1378  oNQ1379  oNQ1380  oNQ581  oNQ582 | Forward - >3kb upstream of twk-16  Nested to oNQ1377  Reverse – Binds in 2nd exon of twk-16. Has *frpr-4* tail  Forward – Binds to start of frpr-4. Has *twk-16* tail  See Above  Nested to oNQ581 | CTTTGTAAGCTATGACGTCACTTC  GACGTCACTTCTTAACAAACGG  GAAAGTCCATCGAATCCATCATTTTTGCATGCTGAAATTTGAAG  GAGTAATACTTCAAATTTCAGCATGCAAAAATGATGGATTCGATGGACTTTC  GAGGTGATGAGGTAGGATGCAAGT |
| **For 3’ RACE of *frpr-4*** | | |
| oNQ549  oNQ550  oNQ551  oNQ578  oNQ579 | Q_T_  Q_o_  Q_i_  *frpr-4* gene-specific primer 1  Nested to oNQ578 | CCAGTGAGCAGAGTGACGAGGACTCGAGCTCAAGCTTTTTTTTTTTTTTTTTGA  CCAGTGAGCAGAGTGACG  GAGGACTCGAGCTCAAGC  ATGTATATCCGTTGTGTATGATGGCAA  GGTATTGATTACTATTGAGCGATGGATTG |
| **£For cloning cDNA of *frpr-4* into pCDNA3.1©** | | |
| oNQ793  oNQ761 | Forward - start of *frpr-4*A  Reverse - stop of *frpr-4*A | CACCATGATGGATTCGATGGACTTTCA  TTATTCGGAAGAAATTCTTGA |
| **§PCR product for *frpr-4* RNAi** | | |
| oNQ627  oNQ628 | Forward – Adds T7 promoter at 5’ end  Reverse – Adds T7 promoter at 3’ end | AATACGACTCACTATAGGGGACTGACGAGCAGAGAAGTTTCG  AATACGACTCACTATAGGGACCTACATATATATCCAGAACAACTC |
